# Uncertainty undermines the validity of antimicrobial pharmacodynamics

**DOI:** 10.1007/s10928-026-10023-0

**Published:** 2026-03-02

**Authors:** Andrew P. Woodward

**Affiliations:** Precision One Health Initiative, College of Veterinary Medicine, University of Georgia, Athens, Georgia USA

**Keywords:** Biostatistics, Bayesian, Antibiotics, Pharmacometrics, Prediction

## Abstract

Antimicrobial therapy is informed by quantitative models of drug disposition and action. These models utilize experimental and observational evidence, subject to uncertainties, to support drug selection and dosage regimen optimization, and interpret antimicrobial resistance data. The framework includes multiple components, which characterize mechanisms contributing to therapeutic outcome. The components must be combined in a logical sequence to generate predictions, so propagation of uncertainty is a critical consideration. Quantitative evaluation of this uncertainty has received apparently little attention. This essay argues for the importance of uncertainty quantification in antimicrobial pharmacology. The impact of parameter uncertainties and measurement errors on the validity of pharmacokinetic-pharmacodynamic modelling of antimicrobials is described. Major components of the modelling workflow are assessed, and uncertainties characterized. The influence of major design and statistical analysis decisions at each step is emphasized. Finally, using detailed simulations, the impact of these sources of uncertainty on outcomes including clinical breakpoints and dose individualization is illustrated. Measurement of antimicrobial potency as the minimum inhibitory concentration contributes approximately twofold error, which is important for individual dose determination. Interpretation of PK/PD parameters is generally conducted dichotomously as thresholds, which are empirically determined, and subject to error. Parameter uncertainties in the exposure-response relationship are potentially substantial, and contribute apparently major uncertainty to predictions at both population and individual levels. The importance of uncertainty in pharmacokinetics appears context-sensitive. Applications including dose optimization or susceptibility breakpoints appear overly confident, and point estimation from these models may be an unreliable basis for decision making. These observations highlight the importance of uncertainty quantification for rigorous antimicrobial pharmacology.

**Supplementary Information:**

The online version contains supplementary material available at 10.1007/s10928-026-10023-0.

## Introduction

Mathematical and statistical models are key tools for both scientific study and clinical applications of antimicrobials [1]. Predictive models of antimicrobial action have obvious applications in dose determination, but also provide support throughout antimicrobial pharmacology, including in the interpretation of antimicrobial resistance data and setting of breakpoints, drug development for neglected or emerging infectious diseases, and translation of in vitro and animal models. The breadth of application of these models, and reasoning that depends upon them, makes their internal validity a critical concern. A major validity threat is propagation of uncertainty; if a dosage recommendation for example was generated by prediction from a mathematical model, and a necessary input variable to the model was subject to measurement error, the dosage recommendation must also be subject to that measurement error [2].

In the clinic, especially for severe or complex infections, information about the individual patient may be explicitly used to determine not only the antimicrobial drug selection, but also the dosage and frequency of administration, under the belief that this is likely to lead to a better clinical outcome [3]. This implies a causal relation between the outcome and the decision process. Clinical trials to test this proposition have been limited. Ewoldt et al. [4] obtained evidence against a meaningful benefit of model-informed precision dosing (MIPD) on either the clinical outcome, or on the achievement of the targeted antimicrobial exposure. The authors emphasized mechanistic reasons that may explain the apparent lack of benefit. However, all the inputs to the computation are based upon empirical evidence, so are uncertain; the more uncertain these inputs, the less informed the decisions based upon them, so the lack of benefit may be explained simply by unreliability of the process. Without quantitative analysis of those uncertainties, their impact cannot be known.

This form of uncertainty is characterized as ‘technical uncertainty’ in the taxonomy posed by Gustafson and Rice [5]; the uncertainty arising as part of the conduct of the scientific process, which may be expressed quantitatively in terms of probability or error. This is distinguished from the inherently provisional nature of scientific findings, disagreement among experts, or lack of necessary background knowledge. In their systematic review [6], communication of technical uncertainty generally had a positive impact on the perception of scientific reporting, consistent with the expectation that individual scientific studies have recognised limitations which are important to quantify. This technical uncertainty, especially in formalized statistical modelling, has often been broadly divided into epistemic uncertainty, which arises from a lack of knowledge and could in principle be overcome by collecting additional or better data, and aleatoric uncertainty, which arises from the irreducible randomness of the system under study. The existence of aleatoric uncertainty is questionable [7] as ultimately all randomness reflects a lack of knowledge; in pharmacokinetics for example, between-subject variation can be explained by introducing covariables, shifting a random feature to a systematic one. The distinction remains practically useful in statistical modelling, so the terminology will be utilized throughout this essay.

Antimicrobial pharmacodynamics necessarily involves a sequence of component models, and each component is subject to uncertainty from various sources, so uncertainty may pass from earlier to later components, and to target predictions. Modelling workflow in the discipline has focussed primarily, or exclusively, on point estimation. For example, in the determination of probability of target attainment (PTA), predictions of the population drug exposure are compared to target values of indices such as the ${AUC}_{24}:MIC$ [8]. Targets are generated empirically from observational or experimental data, as they have no mechanistic basis. Though the exact definition varies between statistical methods, a point estimate corresponds to a single value of a parameter that is most supported by the available information [9]. The available information is never sufficient to allow for perfect knowledge, so without a representation of uncertainty such as an interval, point estimates alone may be misleading [10], and their uncertainty propagates to any function to which they are an input. Confidence intervals for PTA estimates were illustrated by Colin et al. [11], who noted that uncertainty statements were rarely included in published empirical studies of antimicrobial pharmacodynamics. However, it is important to recognize that confidence intervals only describe sampling error (uncertainty arising from intrinsic variability of the data-generating process), and in their illustrated case, only that associated with the pharmacokinetic model. There are other contributors to the overall uncertainty of model predictions [12]. A target index such as the ${AUC}_{24}:MIC$ is itself subject to uncertainty, as it is obtained empirically from finite data. Using only a point estimate of that index to generate the PTA would result in overly confident predictions, as its uncertainty would be ignored.

Various statistical approaches enable quantification of uncertainty, parameter estimation in the presence of multiple uncertainty sources, and simultaneous analysis of multiple related models. From a completed and parameterized model or sequence of models, sensitivity analyses may be useful to quantify the influence of varying inputs on key model outputs, or the importance of key modelling decisions, without demanding statistical tools beyond those already used to conduct standard analyses. However, this process is essentially a tool to explore some of the implications of specified uncertainties, rather than a general and consistent approach to statistical inference [13]. Richer statistical modelling techniques, such as multiple bias modelling [14], facilitate the inclusion of uncertainties as prior information, with correction of resulting estimates. A Bayesian implementation is a natural extension, especially in cases where simultaneous analysis is required across multiple component models, as a joint model inherently allows for propagation of uncertainty between stages. In a generative approach to statistical modelling, fully probabilistic representations of relevant processes are embedded in a narrative description of the system at hand [15], which is a natural framework to embed pharmacologic knowledge in analysis. Modern Bayesian computing tools offer the opportunity to efficiently implement such models for statistical inference [16]. Various related disciplines provide useful illustrations. These ideas have been applied in numerous disciplines, with examples in cell biology [17], physiology [18], and logistics [19]. The importance of uncertainty and its propagation has been long recognised in risk assessment modeling [20], in which potentially many uncertain inputs must be combined. Applications in epidemiology are natural, but utilization has been relatively limited [21]. Various other workflows in pharmacology are being recognized to be subject to similar uncertainties; for example, limitations of observation constrain the estimation of additive low adverse effects [22].

Without adequate consideration of uncertainty, the evidential basis of antimicrobial pharmacodynamic modeling is overstated. Though antimicrobial therapies may often be effective in practice, an inaccurate or incomplete quantification of evidence undermine the scientific foundation of antimicrobial therapeutics, as decisions made with reference to formal models may be less supported by evidence than they appear [23], This essay argues for the importance of uncertainty quantification for reliable inference in antimicrobial pharmacology. To support this argument, the quantitative impact of key model components on parameter estimates and resulting predictions will be illustrated. Analyses will emphasize simulated data inspired by the form of influential studies and systems of practical importance. Though various limitations of the standard approaches to antimicrobial pharmacodynamics have been widely described elsewhere, especially in regard to mechanistic weaknesses [24] and the representativeness and validity of measures [25], this paper will focus on a distinct aspect; the quantification of evidence under the classical workflow, and its implications. Although this has potential clinical importance, especially where decisions are supported directly by model-based reasoning, the worked examples are intended solely to be illustrative of the process, not to imply any specific clinical recommendations.

## Measurement of drug susceptibility

Ultimately the target of antimicrobial agents is a pathogen, and the patient or host is simply the environment in which drug-pathogen interactions take place. Examinations of drug-pathogen interactions, typically in vitro, are a natural starting point for antimicrobial pharmacodynamics, and may generally be taken as independent of patient or host factors. As drug concentration in vivo must be interpreted with reference to drug-pathogen interactions, uncertainty at this stage may propagate through the entire pharmacodynamics workflow.

Potency of antimicrobial effect is usually summarized by the minimum inhibitory concentration (MIC), the lowest concentration, usually from a twofold dilution series, that prevents visible microorganism growth under constant exposure. While other summaries of antimicrobial effect are available, such as the minimum bactericidal concentration (MBC) and mutant prevention concentration (MPC), the MIC is the predominant general-purpose measure. While well-established and fairly standardizable, it offers only a narrow perspective of the pathogen-drug interaction, because the time-course of antimicrobial effects are ignored [26]. The MIC is an outcome of the underlying mechanism, rather than a fundamental feature [27]. Though the measure is simple, practical limitations contribute intrinsic error [28] which may limit its utility.

**Fig. 1:**
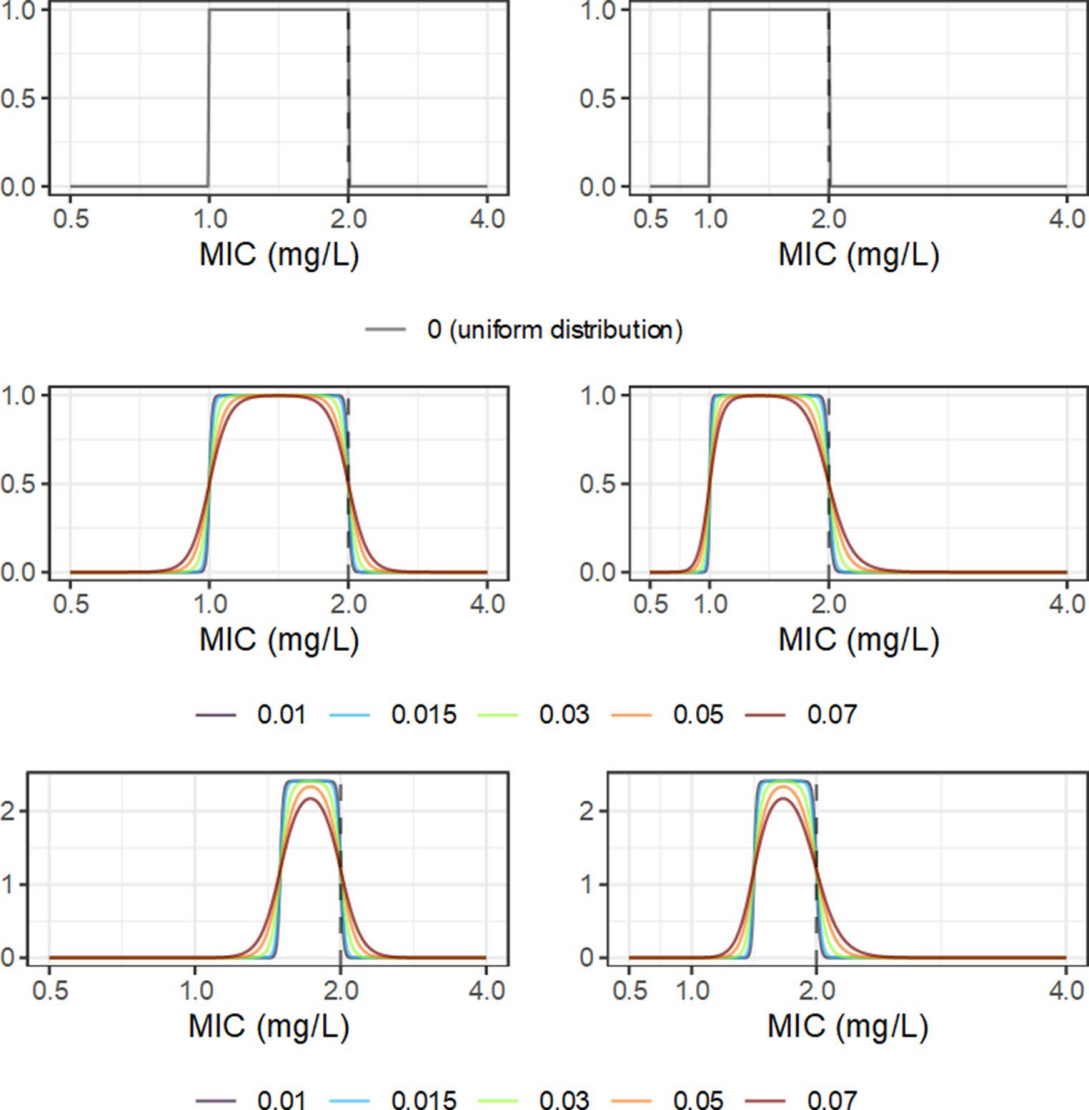
Candidate probability density functions (PDF) for the intrinsic uncertainty of a minimum inhibitory concentration (MIC) observation with a nominal value of 2mg/L, on the log_2_-scale (left) or linear scale (right). The top panels illustrate the uniform distribution with upper limit 2 and lower limit 1, corresponding to the interval censoring implied by twofold dilution. The middle panels represent the logistic mollified uniform distribution with upper limit 2 and lower limit 1, with various values of the logistic component scale. The lower panels represent the LMU distribution with upper limit 2 and lower limit 1.5, corresponding to a dual twofold dilution scale. In all panels the y-axis is the probability density

From the usual microdilution procedure, MIC data are interval censored [29], meaning that the reported value of an observation can only be stated to be between some limits. Though the nominal MIC may in fact be the true MIC, any value between it and the next-lowest-tested value is equally compatible with observation, because concentrations within that range were not tested. If the isolate is only measured once, or if information from technical replication is not somehow formally utilized, then it must be assumed that any value within the interval is similarly plausible. For a single measure, on a twofold dilution series including 1, this could be formalized as the uniform distribution $U\left(2^{(\log2(MIC)-1)},\;MIC\right)$, expressed in Fig. 1.

A minor issue is that values outside of the boundaries are impossible under the uniform distribution (probability 0) but cannot be excluded entirely in practice. For example, a nominal MIC of 2 cannot be taken to indicate that it is impossible that the true MIC is in fact larger than 2, because the tested concentration is itself subject to error; some inaccuracy in preparation is unavoidable. Empirical estimation of such errors in microdilution experiments would be difficult, as they are probably small relative to the dilution scale, so would be swamped by the interval censoring. Presuming good technical conduct, it seems unlikely that the true MIC could be outside the nominal MIC boundaries by more than a few percent. This feature is probably trivial with respect to the overall error, but practicality of computation is also important; the uniform distribution is not continuously differentiable, which may be problematic in parameter estimation. A candidate model for this situation is a mollified uniform distribution [30], a composite distribution combining a uniform distribution with a symmetric continuous distribution. This distribution has an approximately uniform density, but with smoothed ‘softened’ edges. The unboundedness has additional benefits for practical computation when used as a prior distribution. Following [30], the logistic distribution with a small scale is a reasonable choice. Parameterization of the probability density, cumulative distribution, and quantile functions, in terms of the scale $C$ and the upper and lower limits of the uniform component, is expressed in Appendix 1. The selection of *C* for practical applications is mostly subjective, as there is no clear way to estimate it directly; here, the value 0.05 will be used throughout.

It is important to note that this model does not indicate how a MIC observation is related to its true value; rather, it is a representation of the intrinsic uncertainty of the nominal MIC. In other words, in the absence of any other source of error, this is (approximately) the maximum possible precision of a stated MIC value. The total uncertainty is also influenced by other factors, such as between-laboratory or between-operator variation, and potentially bias. For example, well density is evaluated subjectively by the operator, and operators presumably differ somewhat in their judgment of the minimum density that constitutes ‘growth’; small variations in this threshold may cause a whole step of variation on the twofold dilution scale. Several studies have attempted to evaluate between-laboratory variation in MIC data, generally concluding that deviations within one to two twofold dilutions are consistent with random error [31], but examples with apparently greater variation are described [32]. However, quantification of variation in MIC data, obtained under typical conditions, is technically difficult due to interval censoring, and analysis via linear model-based methods as in Mouton et al. (2018) are of suspect validity due to violations of distributional assumptions. Various experimental factors have apparently systematic effects on measured MIC, such as inoculum size [33], which presumably contribute to random variability if uncontrolled.

Surrogate measures of MIC, including disk-diffusion tests, are popular in clinical laboratories [34]. The assay response in the disk-diffusion test, the diameter of the inhibition zone, is an approximately continuous variable, but lacks any direct interpretability in terms of drug potency and must be calibrated with reference to a microdilution-based standard [35]. As the results of disk-diffusion tests are typically interpreted and communicated only in terms of breakpoints, which are themselves model-based estimates which will be considered later, their measurement performance is complex and cannot be viewed in isolation.

In the clinic, the intrinsic error of a single microdilution-based MIC measure contributes, minimally, about twofold uncertainty. For example, if a *Staphylococcus aureus* isolate from a sampled human patient has a reported MIC of 2 mg/L, under the model above it corresponds approximately to the distribution $2^{LMU(\log2(1),\log2(2),0.07)}$, with lower limit 1, upper limit 2, and scale $C$ 0.05. That uncertainty necessarily propagates to any derived measure. Presuming that in the treatment episode this patient’s vancomycin AUC after intravenous administration could be perfectly known (which is too optimistic, as will be discussed later), the target measure, the 24-hour AUC: MIC ratio (${AUC}_{24}:MIC$), can only be known within about twofold error. If for example that patient’s measured 24-hour AUC was 200 mg∙hr/L, the resulting ${AUC}_{24}:MIC$ would be:

1 $$\:{AUC}_{24}:MIC\sim200/{2}^{LMU\left(log2\right(1),log2(2),0.05)}$$

Where $LMU$ is the logistic-mollified-uniform distribution, as above. As the MIC here is represented as a distribution, rather than a single number, and its value could feasibly be anywhere between about 1 and 2 mg/L, the ${AUC}_{24}:MIC$ is also a distribution, denoted by the ‘~’ operator. The resulting distribution for the ${AUC}_{24}:MIC$ has median (a candidate point estimate, though the distribution is fairly flat) 141.4 h, but implies about a 0.95 probability that the ${AUC}_{24}:MIC$ was between 98.5 h and 203 h. If instead the nominal MIC were used, this estimated ${AUC}_{24}:MIC$ would be 100 h. This distribution is visualized in Fig. 1. In this context the intrinsic uncertainty of MIC results in a ‘conservative’ error, i.e. the antimicrobial activity of a given exposure would probably be understated by the point estimate.

This inherent error is a direct consequence of the measurement technique, so is not reducible without modifying it. The width of the uniform component of the proposed model directly represents the interval censoring behaviour arising from twofold dilution, which suggests the possibility of reducing intrinsic error by modifying the dilution series. Typically the twofold dilution series includes 1, but there is no reason that this is required. Consider an MIC determination using additional parallel series [28]; one conventional series including 1, and one including 3; this combined series could be read equivalently to the conventional single twofold series, but its inherent error is approximately 1.5-fold rather than 2-fold. This could be further extended to an additional series including 5, in which case, the error is now approximately 1.2-fold, rather than 2-fold. Though a finer dilution series may bring practical challenges due to cost, standardization, and laboratory workflow, this may be justifiable for the information gain.

## Parameter uncertainty in pharmacokinetics

The rich data and fine control provided from traditional pharmacokinetic studies supports precise estimation of parameters that are inputs for antimicrobial pharmacodynamics. This suggests that in preclinical research, uncertainty in pharmacokinetics may be of limited impact. However, in practical settings, the potential for uncertainty is substantial. Sampling limitations from laboratory animals may prevent the researcher from obtaining sufficient observations for precise knowledge of individual PK. Population pharmacokinetics using multilevel modelling has been instrumental for developing PK predictions for clinical application, but population parameters, especially variability, are difficult to estimate. For dose customization in clinical situations, patient samples may be obtained for therapeutic drug monitoring (TDM), but the resulting inference of the subject-specific PK is necessarily uncertain because of the small number of observations. These examples suggest that uncertainty in PK may enter at many points of the antimicrobial pharmacodynamics workflow.

## Pharmacokinetics at the population level

Uncertainty quantification has been a typical part of the population pharmacokinetics workflow, especially regarding parameter uncertainties. As the pharmacokinetic parameters, represented in the multilevel modelling case by their population distributions, are estimated from finite data, there remains epistemic uncertainty in their value, and therefore in any prediction generated from them. For dose determination, pharmacokinetic parameters are relatively abstract, as drug effect is a function of drug exposure; the utility of the model and its parameters is in making informed predictions of the concentration-time relationship. Epistemic uncertainty in the parameters propagates to predictions, which reflects intuitively that the more information available, the more precise predictions can be made.

Incorporating parameter uncertainty in predictions from multilevel nonlinear models poses technical challenges; for example, while sampling error of predictions may be generated from the Fisher Information, these assume normal sampling distributions, which may be dubious, and though likelihood profiling does not require this assumption, it is only well-defined for the primary parameters and cannot easily be applied to predictions. For antimicrobial applications, generation of confidence intervals via bootstrapping from a population pharmacokinetic model was demonstrated by Colin et al. [11]. Pharmacokinetics has mostly operated under the frequentist paradigm, but its conceptualization of uncertainty in terms of data probabilities is unintuitive and restrictive. The need to quantify and manipulate uncertainties clearly warrants Bayesian statistics. Bayesian estimation via MCMC naturally facilitates prediction uncertainty, as the posterior probability of all the parameters are modelled jointly, which can be extended to functions of the parameters. In this workflow, uncertainty quantification of the predictions follows directly from the joint posterior distribution of the primary parameters.

A case study was generated by simulation, based on the presentation of Kato et al. [36] who described pharmacokinetics of amikacin. The simulated data were intended to emulate those reported by Kato et al. [36] for the purpose of illustration, rather than reproduction. Three simulations were generated to illustrate the relative impacts of both epistemic and aleatoric uncertainties on key predictions from the pharmacokinetic model. As these simulations were based on independent sampling, their results include sampling variation, and can be interpreted as though they were three separate hypothetical studies. In each case, the true locations (population median) of the lognormal distributions of the pharmacokinetic parameters were 40mL/min for plasma clearance, 116mL/min for intercompartmental clearance, 10.7 L for central volume of distribution, and 7.7 L for peripheral volume of distribution. In the first simulation (A), 25 subjects were simulated, which were drawn from population distributions of the pharmacokinetic parameters with true SD (between-subject variation) 0.3. The second simulation (B) had a larger sample size (100 subjects) but was otherwise identical (except for the influence of sampling variation). The third simulation contained 25 subjects, but with greater between-subject variability (SD: 0.6) for all parameters. Each subject received 500 mg of amikacin administered intravenously over 30 min. Observations were then simulated over 24 h following administration, emulating dense sampling, and the amikacin concentration perturbed by proportional error. For verification, the sample distributions of the simulated parameters are visualized with the true simulating distributions (supplementary Fig. 1).

Generation of the statistical models based on the simulated data was conducted in Stan (v2.38) [16, 37] via R (v4.5.2) [38], based upon code described earlier by the author [39]. The two-compartment pharmacokinetic model (the correct model) was assumed in advance. Priors were intended to be weakly informative, presuming minimal knowledge; the key priors were for the clearance, $\:N\left(\mathrm{4,2}\right)$ on the log-scale, which denotes that the value for an average subject was probably between about 7.39 and 403 mL/min, and for the scale of the between-subject variation for all the parameters, which had standard deviations $\:HN\left(0.5\right)$, which is relatively wide and spans a negligible to a high degree of variation. The prior model and MCMC algorithm settings were identical between models. Overall, the parameter estimation was uneventful and the goodness-of-fit of all models was reasonable, based on simple residual analyses (supplementary Fig. 2) and the individual-level predictions (supplementary Figs. 3–5).

The AUC and C_MAX_ are of interest in clinical usage of amikacin [40], so are convenient secondary parameters to summarize the concentration-time function. For each model, a predictive distribution for each of the secondary parameters AUC_∞_ and C_MAX_ was generated by drawing, for each MCMC sample, a single random subject from the population distributions of the PK parameters. The resulting distribution across the samples expresses knowledge of the plausible amikacin exposure, taking into account both the between-subject variation (via the randomly-drawn subject) and the uncertainty in the pharmacokinetic parameters (via the MCMC sampling). Then a tolerance distribution was drawn for each secondary parameter, in which the 10th percentile of 1000 simulated subjects was obtained for each MCMC sample; the resulting distribution across the samples can be interpreted as an expression of the epistemic uncertainty in the lowest amikacin exposure that is reasonably expected in the population under study (the selection of the 10th percentile is mostly arbitrary).

**Fig. 2:**
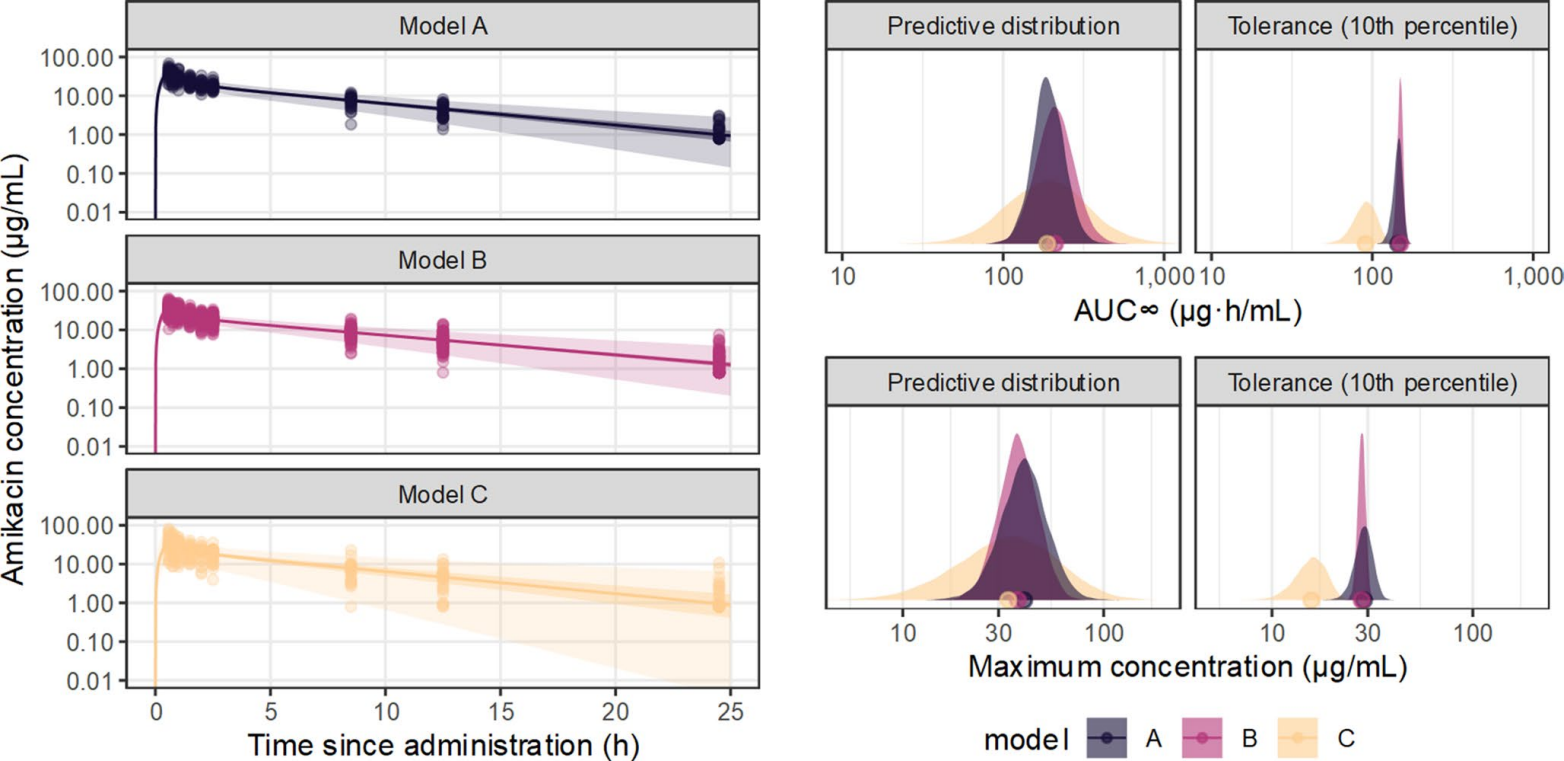
Posterior predictions of amikacin pharmacokinetics from simulated datasets, where amikacin was administered by intravenous infusion over 30 minutes, emulating Kato et al. [36]. The left panel illustrates the posterior-predicted concentration-time function for a hypothetical median subject (ignoring between-subject variation) receiving 500mg amikacin IV as the solid line, the uncertainty (90% credible interval) as the central field, and the 90% posterior predictive distribution (encompassing between-subject variation) as the light field. Model A had 25 subjects with moderate between-subject variation, Model B had 100 subjects with moderate between-subject variation, and Model C had 25 subjects with high between-subject variation. The right panel shows the posterior predictive distribution and tolerance distribution (of the 10th percentile of hypothetical subjects), of the AUC_∞_ and C_MAX_, points are posterior median

**Fig. 3:**
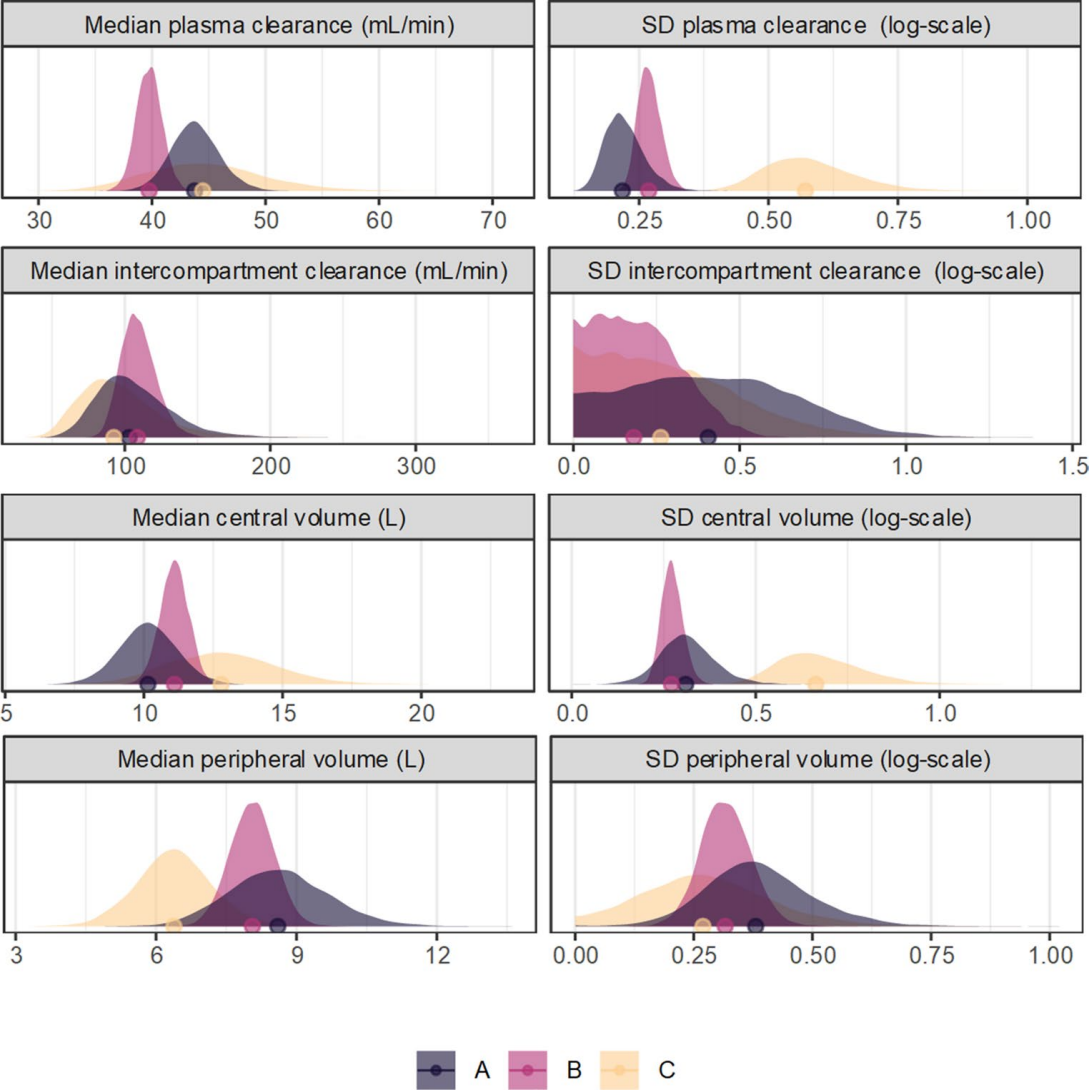
Marginal posterior distributions for the primary pharmacokinetic parameters from models based on simulated data designed to emulate the pharmacokinetics of amikacin as described by Kato et al. [36]. Model A had 25 subjects with moderate between-subject variation, Model B had 100 subjects with moderate between-subject variation, and Model C had 25 subjects with high between-subject variation. Points represent the posterior median

The resulting posterior predictions are summarized in Fig. 2. From the posterior predictions of the concentration-time function, the decreasing uncertainty in the expectation for a hypothetical average subject is shown by the width of the credible region, which was narrowest for model B (with the largest sample size) and widest for model C (with the largest between-subject variation). The prediction region, expressing both parameter uncertainty and variation, was of similar width between models A and B, suggesting that the total uncertainty in the expected exposure for a new subject was driven mostly by between-subject variation. The prediction region for model C was accordingly wider. These patterns are reflected in the posterior distributions for the population primary parameters (Fig. 3), which demonstrate overall that both the locations and scales of the parameters were most precise for model B, demonstrating the reduced epistemic uncertainty from the larger sample size, and the between-subject variation was generally largest and most uncertain in model C.

Similar patterns are apparent in the posterior distributions of the AUC and C_MAX_ (Fig. 2). The predictive distributions were similar between models A and B, suggesting that the expected amikacin exposure for any future subject was mostly uncertain due to between-subject variation, rather than parameter uncertainty, which was also reflected in the wider predictive distribution for model C. However, the expectation for a fixed proportion of future subjects was substantially more uncertain for model A than for model B, demonstrating the benefit of the larger sample size. The lower value (shifted left) of the tolerance distribution for model C demonstrates the influence of the larger between-subject variation, as more extreme subjects are plausible under this model. However, the increased between-subject variation, without an increase in the sample size, resulted in greater parameter uncertainty for model C, consistent with the greater width of the tolerance distribution. In each case, a point estimate (posterior median) is visualized to demonstrate the extent of information loss implied by ignoring the parameter uncertainties.

These effects have direct implications for the usage of pharmacokinetic models as inputs in pharmacodynamics. If for example the 10th percentile was applied as an indicator of the target exposure, rational decision making should consider how much is known about that estimate given the available information, not simply its ‘best’ value. Further, the degree of uncertainty is dependent on the design and will therefore vary between studies, and this differential uncertainty would be hidden if only point estimates were used, which would be particularly impactful for studies with a small sample size or other sparsity problems. Consumers of such studies may be overly optimistic about their informativeness, or generate clinical conclusions that are not well-supported by the available evidence. For example, if a small pharmacokinetic study was conducted in a special patient population, then the support for a dose adjustment or other decision must be judged with reference to the uncertainty in the target exposure relative to a reference condition, not simply a point estimate, as epistemic uncertainty may be substantial.

## Pharmacokinetics at the individual level

Reasoning from population pharmacokinetic models, using predictions such as the probability of target attainment, encapsulates between-subject (and potentially additional levels of) variability. Though a well-performing model may provide strong inference regarding plausible future individuals, it may provide little or no additional insight about any specific individual. It is widely held, and intuitive, that obtaining individual-specific data in the form of therapeutic drug monitoring (TDM) may aid decision-making for antimicrobial therapies [41], especially in conjunction with statistical models for model-informed precision dosing [42]. However, individual PK must be inferred as the available information is necessarily incomplete, so is subject to uncertainty. However, the uncertainty of individual MIPD information is not addressed in recent discourse [43].

Individual parameters have been widely obtained by maximum-a-posteriori (MAP) estimation [44]. This may be considered a form of Bayesian optimization in which a point estimate, ostensibly a single best value, corresponds to the mode of the posterior distribution. Though this is easier to obtain than the complete posterior distribution, it does not indicate the extent of uncertainty in the resulting PK estimates. While the MAP corresponds to the ‘most supported’ single value in some sense, it does not indicate *how much* more supported it is by the available information than any other candidate value. In the oncology context the implications were recently addressed Maier et al. [45]. While other aspects of MAP estimation for antimicrobial applications have been addressed, such as their accuracy [46], the bias incurred by shrinkage [47], and the weight of priors [48], their uncertainty appears to be largely unaddressed. Technical barriers may explain why full posterior probabilities, which are key to Bayesian inference, are not generally a part of MIPD communication, though the process is often explicitly described as Bayesian. Recent developments involving data assimilation [49] implement full posterior inference for new subjects, which allows for dose optimization informed by individual uncertainty; these have emphasized oncology applications, but the same benefits are clearly relevant for antimicrobials. As the uncertainty of individual PK estimates are probably quite context-dependent, no single example is likely to be illustrative, so this is not further explored here.

## Uncertainty in pharmacokinetic/pharmacodynamic indices

Seminal work in animal models was instrumental in establishing quantitative relationships between drug exposure and antimicrobial drug effects in vivo. Pivotal findings in rodent models [50] demonstrated evidence that the outcome of treatment with fluoroquinolone antibiotics was more strongly predicted by the plasma concentration area-under-the-curve (AUC) than the maximum plasma concentration (C_MAX_) or time-above-MIC (T > MIC), while for aminoglycosides the outcome was most strongly predicted by the C_MAX_ [51]. These measures are substantial simplifications of the concentration-time relationship, so cannot be representative of the underlying data-generating process, which would link the outcome to the entire concentration-time function. More mechanistic models including real-time effects on pathogen dynamics have been explored for antibacterial agents in vitro [52], and for a number of special antimicrobial applications in vivo, such as treatment of malaria [53], HIV [54], and tuberculosis [55]. However, for general antibacterial applications the classical ‘index’-based approach has been predominant, so that will be the focus here. These are empirically motivated statistical features that represent the complex unobservable interactions that control the outcome of infections. Without a mechanistic foundation, the rigor of these models depends strongly on the quality and amount of the observational data, and on the statistical decisions that are made to generate them.

### Exposure targets are uncertain

Observational studies have been widely applied to evaluate relationships between drug exposure and the outcome of antimicrobial therapies in clinical patients. In principle, these designs provide more direct evidence of the probability of treatment success than animal or in vitro models. Dose determination for vancomycin has emphasized the target $\:{AUC}_{24}:MIC$ 400 h, which originates from analysis of pneumonia treatment outcome in humans [56]. Subsequent studies have evaluated the association between vancomycin $\:{AUC}_{24}:MIC$ and the microbiological or clinical outcomes [57, 58] and generated their own estimated targets. However, in prospective evaluation, achieving an a priori vancomycin exposure target did not apparently confer a meaningful benefit [59]. Uncertainty in the input variables may explain the lack of an apparent benefit.

The AUC: MIC target is empirical, having no underlying mechanistic basis, so it is uncertain. Measurement error in the MIC is one contributor to its uncertainty. Another is the sampling error imposed by the finite sample size. To explore the effects of uncertainty propagation on generating and utilizing an exposure target, synthetic data were simulated to approximate that reported by Moise-Broder et al. [58], but with a larger sample size to ensure computational stability. Specifically, 200 observations of AUC were drawn from the lognormal distribution $\:LN({\mathrm{log}}_{e}\left(400\right),0.6)$, and the true MIC on the *log*_2_ scale from the normal distribution $\:N\left(\mathrm{0,0.8}\right)$, which has median MIC of 1 mg/L, with the AUC and MIC uncorrelated. With the resulting AUC: MIC as the predictor, the probability of a positive outcome was defined by 4-parameter log-logistic model, with minimum success probability 0.2, maximum 0.7, ED_50_ 600 h, and slope *H* 1.5. The final data were expressed as the observed outcome for each subject via the Bernoulli distribution. The simulated AUC and MIC observations are visually expressed as histograms, with the simulating distributions, along with the true dose-response relationship and the simulated success probability by subject (supplementary Fig. 6).

A popular method to obtain an exposure target involves partitioning; divide the data into two cohorts using a threshold value, typically the value that provides for the largest difference in the outcome between the split cohorts. This definition of exposure target has major conceptual and practical flaws. If the objective were to simply predict the outcome given the exposure, then a single number is simply a very inefficient strategy to do so because of information loss, as for dichotomization in other applications [60], and a continuous model would be a better candidate. Further, while the threshold provides an efficient means to separate the data into two cohorts, it is not clear why that is a useful basis for therapeutic decision making. That is, in some sense, statistically efficient, but there is no reason why it corresponds to a sufficient clinical benefit.

**Fig. 4:**
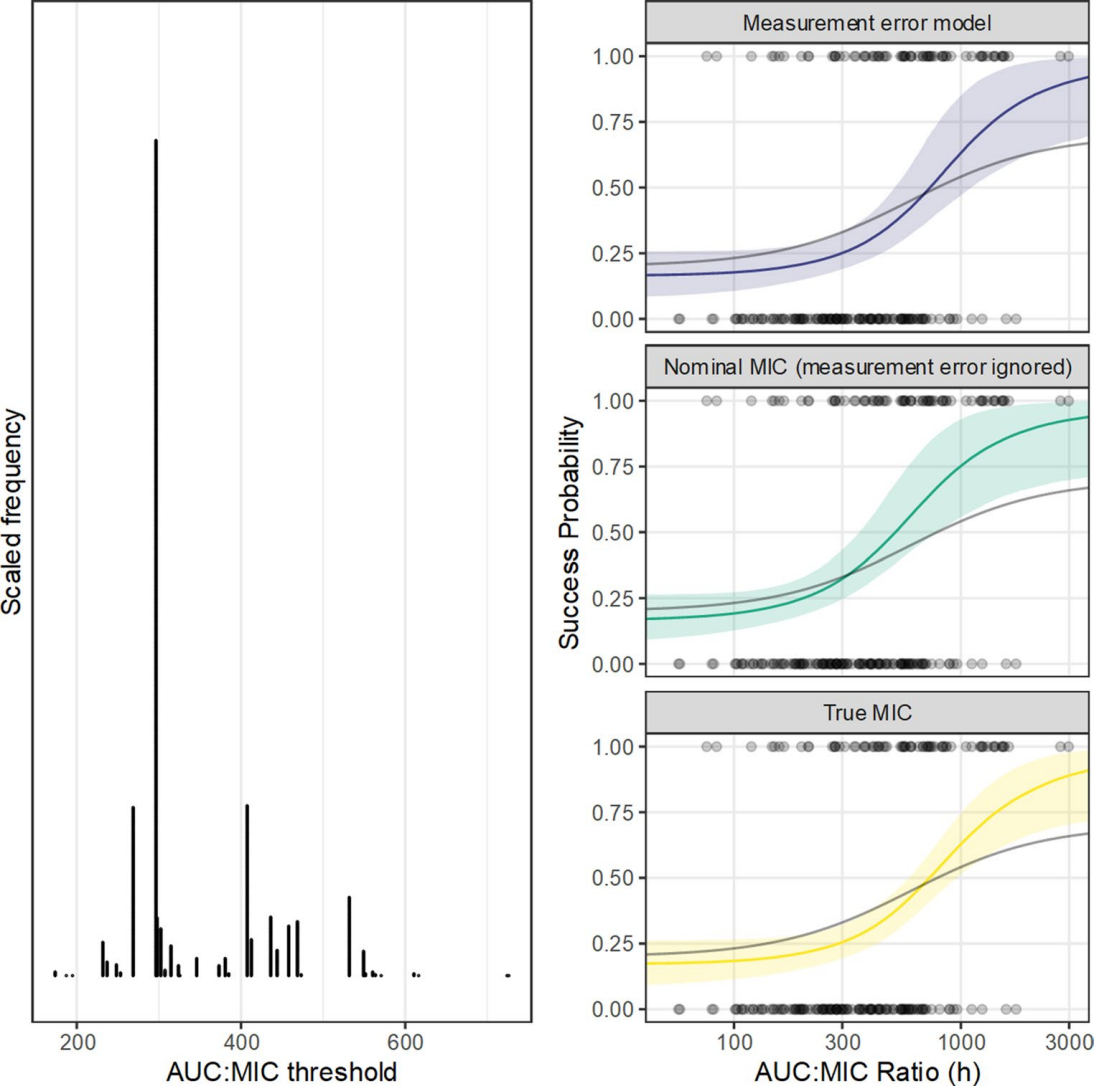
Bootstrap distribution for the ‘optimal’ exposure threshold for vancomycin, generated from simulated data based on the design and results of Moise-Broder et al. [58]. The exposure thresholds were generated via a decision stump assuming that the response increases monotonically with AUC:MIC. The bars are composed of stacked dots representing the count of bootstrap samples (total 2000 bootstrap resamples). The point estimate (627 in this simulation) is the most supported, but there is meaningful support for relatively distant values (including 169, 238, 438, 536). Note that this distribution is discrete (57 unique values) because the statistic is generated by rearrangement of the data at hand, and only finite arrangements are available. Posterior predictions from generalized nonlinear models (4-parameter logistic with Bernoulli outcome) for the probability of a good outcome given antimicrobial drug exposure (AUC:MIC ratio). The underlying data are simulated and were intended to emulate the design and results reported for vancomycin by Holmes et al.[57] and Moise-Broder et al. [58]. The observations are the grey points aligned on 0 and 1 for failures and successes. The black line is the true data-generating function which was linear onthe logistic scale, with the latent MIC (no measurement error) as predictor. The yellow line is the posterior median predicted response from the GLM using the nominal MIC as predictor (ignoring the measurement error), with the prediction made from the latent MIC. The purple line is the posterior median predicted response from the GLM including a measurement error model for the MIC via the logistic-mollified uniform distribution, with the prediction made from the latent MIC. Fields represent 90% credible intervals (posterior quantiles) for the prediction

To illustrate the technical uncertainty implied by threshold-type targets, an estimate was obtained from the simulated data using a decision stump as implemented in package ‘MKclass’ [61], assuming that the probability of a successful outcome increased monotonically. The result 296.2 h is the single value of AUC: MIC that resulted in the largest difference in the outcome between the split cohorts. This value is a point estimate; its sampling error was approximated by resampling [62]. The sampling distribution comprising 2000 bootstrap resamples (Fig. 4) demonstrates that, even with a reasonably large sample size, there is meaningful uncertainty about the apparent optimum threshold. The point estimate 296.2 h is of course the most supported (680/2000 resamples) along with two nearby values 296.4 h and 297 h (314 resamples), but there is meaningful support for distant values including 268 h (166/2000 resamples), 407 h (190/2000 resamples), and 531 (88/2000 resamples). The discreteness of the sampling distribution arises because only a finite number of arrangements of the given data are available. It is important to note that because only a single binary outcome is available for each subject, the simulated data, and therefore the estimated optimal value and the exact form of the distribution may vary substantially between simulations. In practice, sampling variation is an important contributor to the parameter uncertainty; while this can be examined directly in simulations, it is not observable in reality. Variation between simulations was not further examined here, so this simulation corresponds to the form of the results that might be expected from a single hypothetical study.

Actual expression of the uncertainty of exposure thresholds appears to be rare in the literature. These estimates are often accompanied by frequentist hypothesis tests, which has raised some contention [63]. Commonly to the problematic dichotomous interpretation of frequentist hypothesis tests [10], it appears that the hypothesis test is often misinterpreted as a statement of evidence in favour of the point estimate, though it is in fact a statement of evidence against the test hypothesis, and does not convey anything about how well the estimate is known. For example, the statement:

“…an alternative vancomycin AUC/MIC of > 373, derived using classification and regression tree analysis, was associated with reduced mortality (P 0.043)…” [57].

suggests the point estimate 373 is substantially preferable than the value 400, because it was ‘statistically significant’ and the comparator was not; this interpretation is fallacious, as the difference between them is not necessarily substantive [64]. A more reasonable interpretation is that the value of the threshold is uncertain, but the value 373 was most supported by the data at hand. However, this does not indicate how much the available evidence supports this estimate without an expression of the degree of support for other values.

### Beyond dichotomization

A more efficient and rigorous usage of the observational data is to generate a parametric exposure-response model. Compared to the exposure-threshold approach which reduces the relationship to a single number, an appropriate exposure-response model may capture the full functional form. From the simulated data, the probability of a successful outcome was described using a 4-parameter log-logistic model [65], implemented in Stan [16], with a weakly-informative prior model. The relationship was constrained to be strictly-increasing by defining the E_MAX_ to be larger than E_MIN_. To assess the influence of measurement error in the MIC, three models were generated. Firstly the ‘true’ latent MIC, which is the true predictor but unobservable in practice, was used to define the AUC: MIC. Secondly, the nominal MIC (ignoring measurement error) was used as the predictor. Finally, the MIC measurement error was implemented via the logistic-mollified uniform distribution, by defining the true MIC as a parameter to be estimated in the model, presuming a single twofold dilution series. The parameters of the exposure-response model were estimated for each case, and the posterior-predicted exposure-response relationship was visualized (Fig. 4) to express the degree of uncertainty in the probability of a successful outcome. For any AUC and MIC combination, the expected outcome can be obtained from these models along with its uncertainty. In this simulated case, the extent of uncertainty in the exposure-response relationship, which can be observed as the width of the credible region, was reasonably similar between models (Fig. 4). Though this model is more expressive, similarly to the bootstrap estimation, this is just the result from one simulation, and the precise form is subject to meaningful sampling variation. In real situations, this sampling variation is inevitably present.

## Propagation of uncertainty: breakpoint determination

Clinical breakpoints attempt to facilitate the representation of antimicrobial effect information in terms of a prediction of clinical efficacy. As the direct interaction between drug and pathogen represents only, in simple terms, drug potency information, additional context is required to interpret laboratory measures such as the MIC. A breakpoint is a threshold value that purports to indicate when phenotypic resistance is sufficient to predict a poor probability of success. Breakpoints may be determined in a bottom-up approach, by a quantitative or semiquantitative combination of pharmacokinetic and pharmacodynamic evidence [66]. From the pharmacokinetic evidence, drug exposure is predicted, given the dose regimen. The combination of predicted drug exposure with a potency measure, such as the MIC, indicates the magnitude of the expected effect, and the probability of a successful outcome is then predicted based on that apparent magnitude. Finally, the breakpoint is the lowest potency (equivalently, the highest degree of resistance) for which the probability of the successful outcome is sufficiently high, given the exposure at the selected dose.

Breakpoint determination therefore rests on at least three interrelated lines of evidence; the drug exposure expected given the dose regimen (PK), the relationship between drug exposure and antimicrobial effect (PD), and the clinical outcome expected given the exposure. As we have explored, each of these elements is subject to potentially multiple sources of uncertainty, which necessarily propagate to the resulting breakpoint. Similarly to decision analysis regarding the dose regimen, the contextual interpretation of the MIC (or any in vitro potency measure) relies on reference to a PK-PD model, so its uncertainty is dependent on that of the underlying component models. Failure to account for these sources of uncertainty my result in excessive confidence in the resulting breakpoints.

Ciprofloxacin has been widely used in humans [67], but has been less popular in veterinary applications where it has generally been substituted with the prodrug enrofloxacin [68]. In the dog, its oral bioavailability is considered a limiting factor, leading to a comparatively low proposed breakpoint of 0.06 mg/L, presuming a 25 mg/kg dose [69]. This determination rests on evidence of the pharmacokinetics of ciprofloxacin in dogs, and the relationship between ciprofloxacin exposure and the clinical outcome. This is a useful example to illustrate the implications of uncertainty on the determination of clinical breakpoints.

Consider hypothetical pharmacokinetic data, simulated to emulate the pharmacokinetics of ciprofloxacin in the dog as reported by Papich [70] after intravenous administration and Papich [69] after oral administration. To model a typical sample size for a descriptive preclinical pharmacokinetic study, 6 subjects receiving an intravenous bolus dose were simulated, 2 for each of 5, 10 and 25 mg/kg. To emulate the extent of evidence provided by a study of the form of [69], 35 subjects receiving an oral dose of ciprofloxacin were simulated, with the dose randomly selected (with equal probability) of 5, 10 or 25 mg/kg. The population parameters were selected to mimic those studies. All were defined as lognormal distributions, except for the absolute bioavailability, which was logistic-normal, and were uncorrelated. Locations of the population distributions, on the response scale (i.e. the population median), were 587 mL/h/kg for $\:Cl$, 356 mL/h/kg for $\:{Cl}_{Q}$, 1780 mL/kg for $\:{V}_{C}$, 1874 mL/kg for $\:{V}_{P}$, 0.5 h^− 1^ for $\:{k}_{a}$, and 0.6 for $\:F$. All had moderate between-subject variation with scale $\:\sigma\:=0.3$ on the linear-predictor scale, except for $\:F$ which had scale $\:\sigma\:=0.5$. The sample distribution of the simulated subjects are expressed as histograms, with the simulating distributions for verification (supplementary Fig. 7). For each subject, observations were simulated at 10, 20 and 40 min, and 1,1.5, 2, 4, 6, 8, 12, and 24 h after administration (to emulate rich sampling), and proportional-normal residual error added with proportional standard deviation 0.2.

From these data, a population pharmacokinetic model was generated, using a two-compartment structural model with simple first-order absorption (i.e. the correct model), proportional normal error, and between-subject variation in each pharmacokinetic parameter. This is a standard model, but of course in practice would not be known to be the correct selection. Analysis was implemented in Stan [16] via R [71] based on code developed by the author [39], using weak prior information. Details of the fitted model are visualized, including the goodness-of-fit (supplementary Fig. 8) and approximate posterior distributions of the primary parameters (supplementary Fig. 9). The posterior predictive distribution and tolerance distribution (of the 10th percentile) of the AUC as a function of the dose, implied by the completed model, are also expressed (supplementary Fig. 10) which are the basis for the later predictions.

**Fig. 5:**
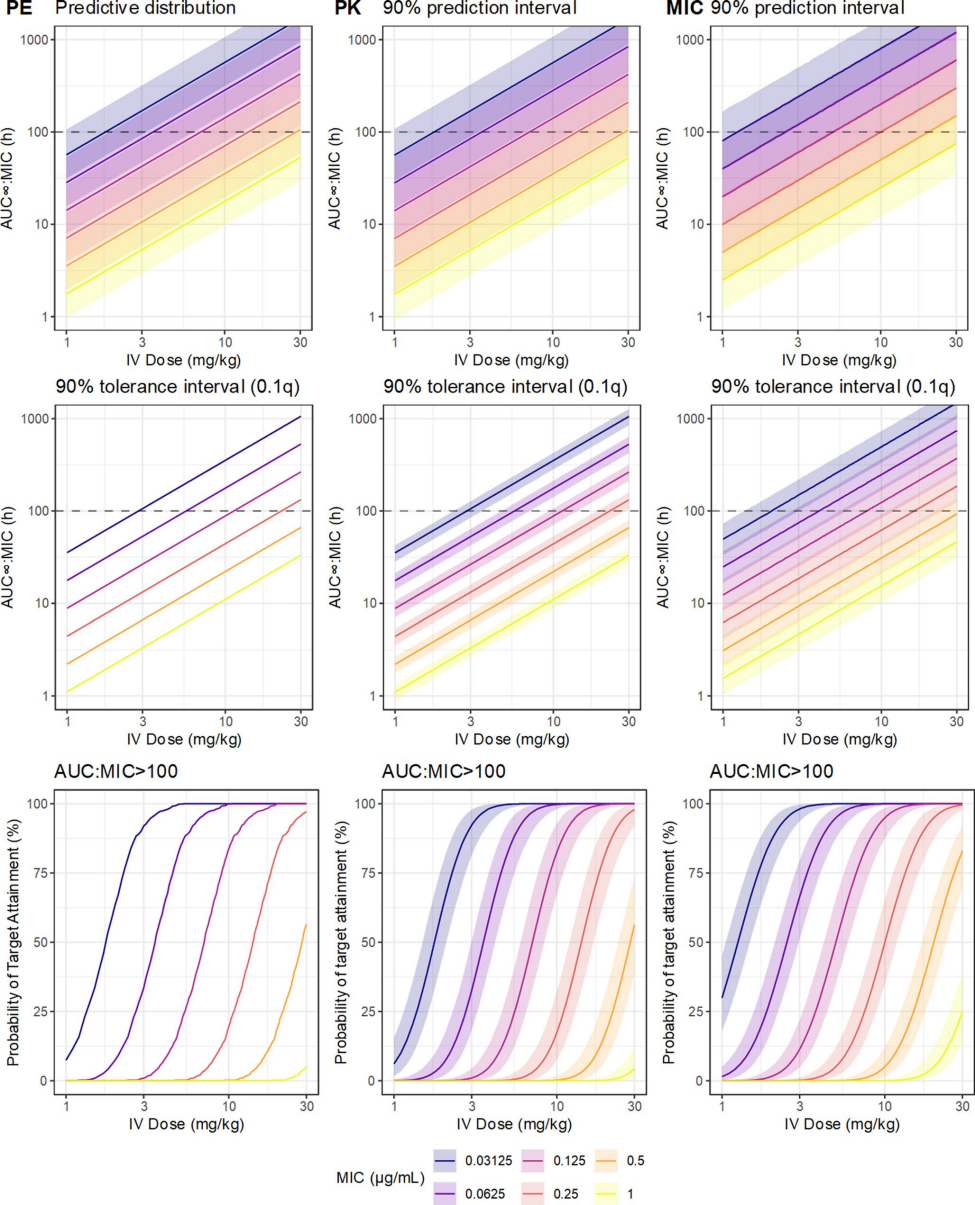
Predictions of ciprofloxacin population pharmacokinetics from the PK model based on simulated data, after IV bolus administration. The top row shows the predictive distribution which denotes the range of ciprofloxacin exposures that are plausible under current knowledge, taking into account only the between subject variation. The middle row shows the 10thpercentile of predicted ciprofloxacin exposures. The bottom row describes the proportion of hypothetical future individuals (probability of target attainment) expected to have AUC∞:MIC greater than the nominal target (100h). The left column (PE) are point estimates (posterior median of each of the PK parameters), souncertainty is ignored.The middle column (PK) adds the parameter uncertainty from the PK model by using the joint posterior distribution of the parameters. The third column (MIC) adds the inherent uncertainty of the MIC measurement, implemented via the logistic-mollified uniform distribution

As no uncertainty statements were presented, the exposure predictions for breakpoint evaluation generated by Papich [69] were apparently from point estimates of the parameters, which is likely the typical method as discussed by Colin et al. [11]. To emulate this reasoning, point estimates of the location and scale of the pharmacokinetic parameters were taken as their posterior median. A simple pharmacodynamic model for the antimicrobial effect is the AUC: MIC ratio, the typical choice for the fluoroquinolones, so the expected population distribution of the AUC: MIC ratio (the AUC_∞_ was used for computational simplicity) was generated by simulation of 1000 hypothetical subjects, at each of a series of candidate values of the MIC (which is taken as fixed and known). The target AUC: MIC for determination of the probability of target attainment was taken as 100 h [69]. The predictions are summarized in Fig. 5, both as the predictive distribution and as the 10th percentile. These point estimates correspond to the most supported predictions given the available information, but ignore all epistemic uncertainty.

**Fig. 6:**
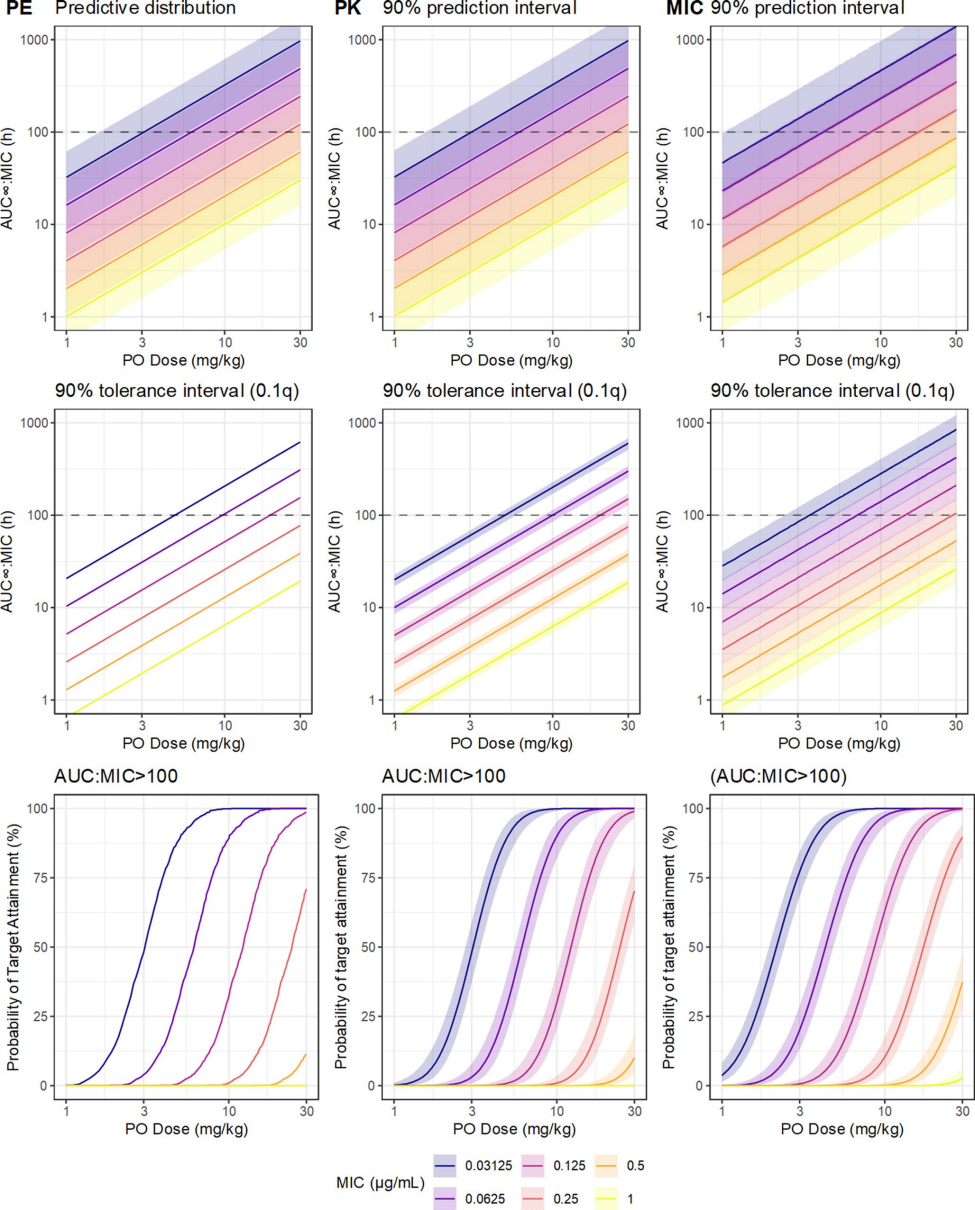
Predictions of ciprofloxacin population pharmacokinetics from the PK model based on simulated data, after single PO administration. The top row shows the predictive distribution which denotes the range of ciprofloxacin exposures that are plausible under current knowledge, taking into account only the between subject variation. The middle row shows the 10thpercentile of predicted ciprofloxacin exposures. The bottom row describes the proportion of hypothetical future individuals (probability of target attainment) expected to have AUC∞:MIC greater than the nominal target (100h). The left column (PE) are point estimates (posterior median of each of the PK parameters), so uncertainty is ignored. The middle column (PK) adds the parameter uncertainty from the PK model by using the joint posterior distribution of the parameters. The third column (MIC) adds the inherent uncertainty of the MIC measurement, implemented via the logistic-mollified uniform distribution

Though the predictions based on the point estimates are relatively intuitive and simple to compute, they are overly optimistic, because they have not taken into account uncertainty in their inputs. An obvious source is the parameter uncertainty in the pharmacokinetic model, which was described in detail by Colin et al. [11]. As the result of the Bayesian analysis is a joint probability distribution over all its parameters, it is quite simple to translate uncertainty of the parameters into functions of those parameters by operating on the MCMC samples. Instead of generating 1000 hypothetical subjects from the only the posterior medians of the parameters, parameter uncertainty is implemented by simulating from the values of the parameters at each MCMC sample; either of one subject per sample, which generates a predictive distribution, or of 1000 subjects per sample, from which a tolerance distribution may be obtained, given a quantile of interest. The predictive distribution, given the MIC and the dose, describing the probable value of new observations [71], and the tolerance distribution, describing uncertainty in the 10th percentile of observations, is shown in Fig. 6. Compared to the point estimates alone, these posterior predictions additionally represent the epistemic uncertainty contributed by the PK model parameters. So far, the uncertainty implied by the tolerance distributions remains relatively narrow, relative to the systematic difference contributed by the MIC, which demonstrates that parameter uncertainty in the PK would have a relatively minor effect on breakpoint determination in this case.

**Fig. 7:**
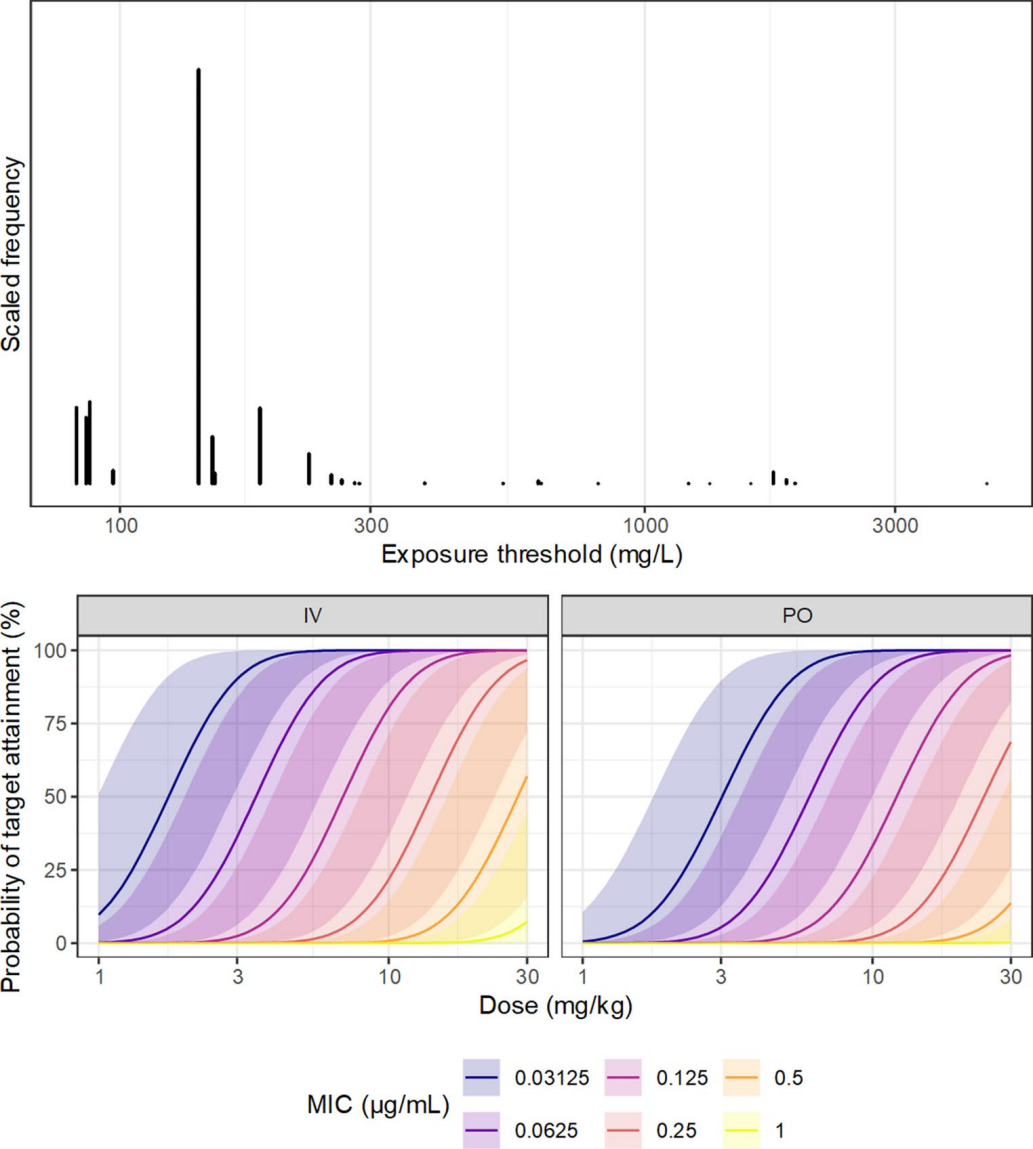
The upper panel shows a bootstrap distribution for the ‘optimal’ exposure threshold for ciprofloxacin, generated from simulated data based on the design and results of Forrest et al.(1993). Exposure thresholds were generated via a decision stump assuming that the response increases monotonically with AUC:MIC, and the sampling distribution summarized by nonparametric bootstrap. Bars are composed of stacked dots representing the count of bootstrap samples (total 10000 bootstrap resamples). The lower panel shows the estimated probability of target attainment as a function of ciprofloxacin dose and MIC, taking into account the uncertainty in PK parameters, inherent uncertainty in MIC, and the bootstrap uncertainty in the AUC:MIC target, from simulated ciprofloxacin PK data based on the design and results of Papich (2012) and Papich (2017). The solid line is the posterior median, and the fields the 90% credible regions for the posterior predictions

The pharmacokinetic parameters are not the only relevant source of uncertainty. Presuming that the MIC is obtained in practice from a single twofold dilution series, the MIC has approximately twofold intrinsic error, which propagates to the PK/PD model. From the nominal MIC, the underlying ‘true’ MIC may be represented by the logistic-mollified uniform distribution, as discussed earlier. At each MCMC sample, and at each hypothetical observation, a further random sample is drawn from the proposed MIC distribution, resulting in predictions of the AUC: MIC that take the intrinsic MIC uncertainty into account. These predictions are expressed in Fig. 7. Notably, these predictions at the population level are not meaningfully more uncertain, as the width of the credible regions is approximately the same as in Fig. 6; however, the predicted AUC: MIC are systematically larger, demonstrating the downwards bias in predictions resulting from ignoring the inherent MIC uncertainty.

Breakpoint determination is typically made by comparison of the population predicted exposure to some pharmacodynamic target. In addition to the parameter uncertainty from the PK and the measurement uncertainty contributed by the MIC, the target AUC: MIC is also uncertain. With the presumption that the uncertainty in the target AUC: MIC and the uncertainty in PK are independent, which seems reasonable if these estimates are generated by separate studies, then their contributions are simply additive. The analysis reported by Forrest et al. [72] has been influential for the AUC: MIC target for the fluoroquinolones. To emulate the parameter uncertainty in a threshold-type target from a single dataset of this size, simulated data were generated from a 5-parameter log-logistic model, with parameters chosen to mimic the data reported by Forrest et al. [72]; the EC_50_ was set as 50 h, the E_MIN_ (at zero exposure) was 0.05, and the E_MAX_ (at infinite AUC: MIC) was 0.85. As in the earlier example, the exposure-response model for simulation expressed the probability of a successful outcome as a function of simulated values of the AUC: MIC, and the simulated binary response for each subject was generated from the Bernoulli distribution. The simulated distributions of the AUC and MIC are expressed as histograms, with the true dose-response relationship with the simulated outcomes, in appendix 1.

From the simulated exposure-response data, the target AUC: MIC was estimated via a decision stump with its sampling distribution approximated by bootstrapping. The bootstrap distribution is expressed in Fig. 7. Presuming that the bootstrap distribution can be taken to represent an approximate posterior distribution for the threshold [73], and that it is independent from the pharmacokinetic model, the posterior distribution for the PTA was obtained by taking one bootstrap sample of the target AUC: MIC for each MCMC sample. The resulting predicted PTA, expressed visually in Fig. 7, takes into account all the sources of uncertainty previously considered, and parameter uncertainty in the ‘floating’ AUC: MIC target. Subjectively, based on visual examination of the PTA functions expressed in Fig. 7, the total uncertainty is clearly sufficient to diminish confidence in a selected breakpoint, shown by the substantial overlap in the posterior predictions between MIC values, and the broad credible region around the transition point implies high sensitivity to alteration of the dose.

**Fig. 8:**
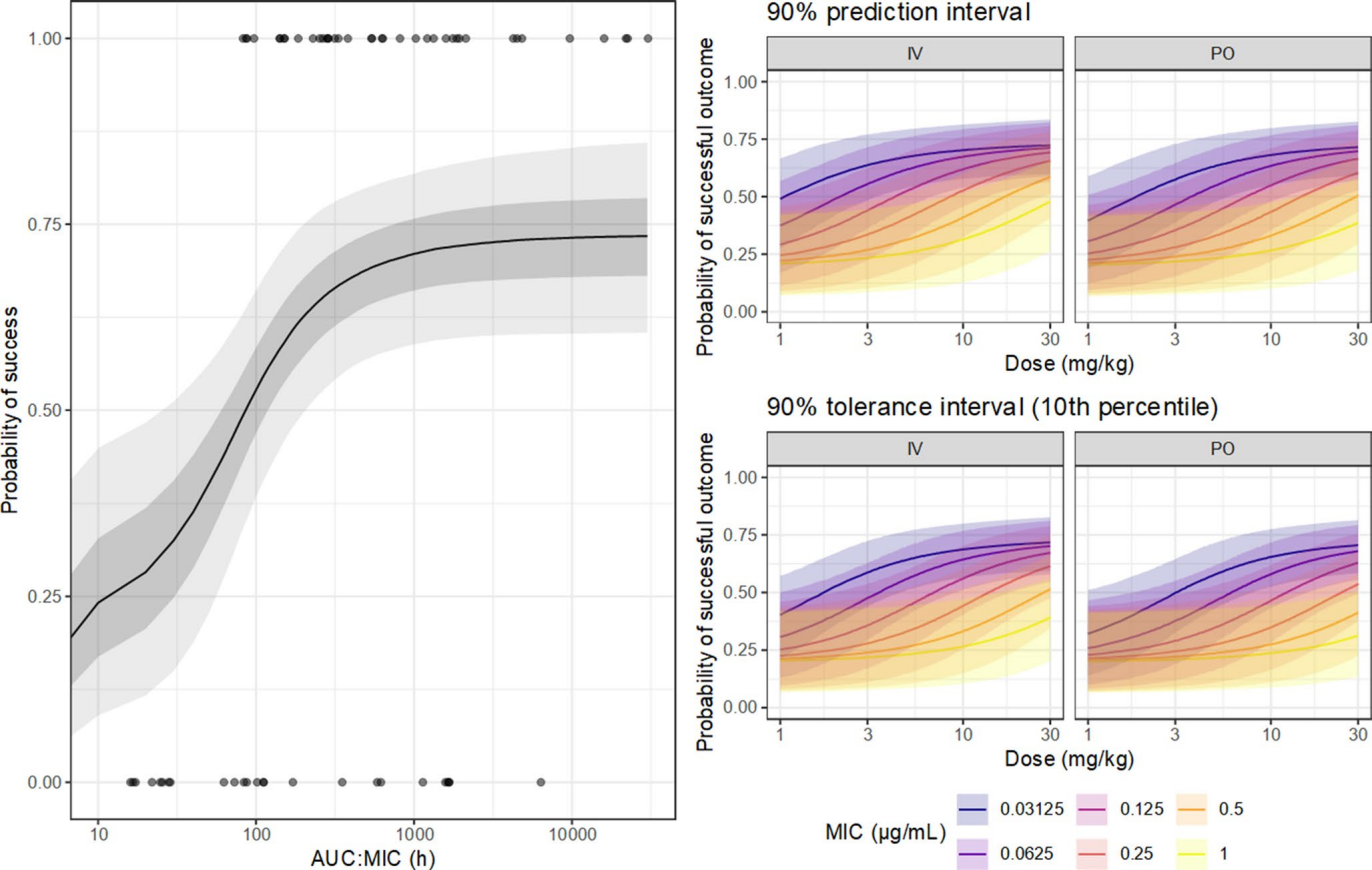
The left panel shows an exposure-response model for ciprofloxacin, from simulated data based on the presentation of Forrest et al. 1993. The points are the observed (0: failure, 1: success) outcomes. The solid line is the posterior median predicted success probability and the fields the 50% and 90% credible regions. The right panels describe approximate posterior distributions of the expected treatment exposure-response relationship, given the dose. Rather than summarize the outcome using an exposure target, the predicted drug exposure for each hypothetical subject is passed to the full dose-response model, for the probability of treatment success at the individual level. The top row describes the predictive distribution of the expected outcome, taking into account parameter uncertainty in both the PK and exposure-response models, the between-subject variation, and the intrinsic uncertainty in the MIC. The bottom row is a tolerance distribution for the expected outcome, expressing the uncertainty in the 10th percentile of predicted outcome probability. The strongly sigmoid form of the predicted relationships reflects the form of the exposure-response component of the model

**Fig. 9:**
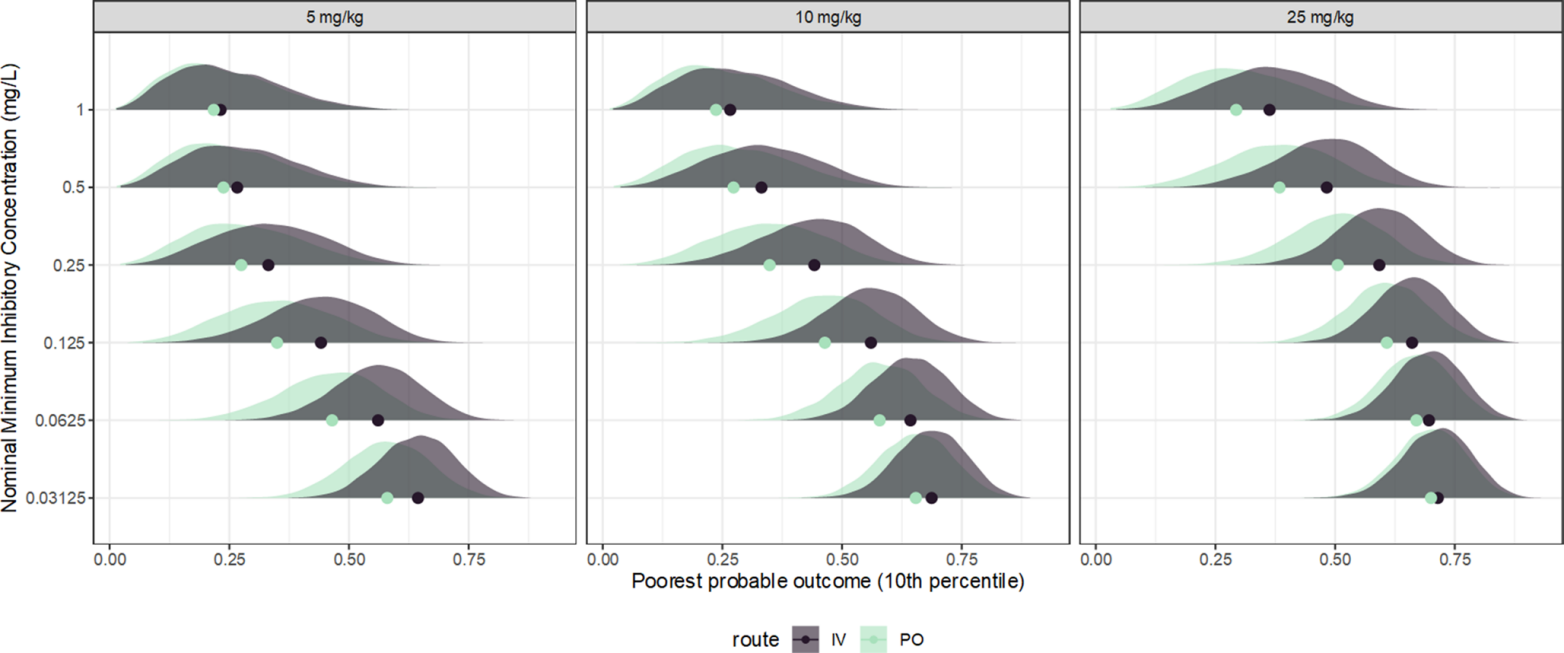
Approximate posterior distributions of the expected treatment outcome, given the treatment regimen and the dose. Rather than summarize the outcome using an exposure target, the predicted drug exposure for each hypothetical subject is passed to a proposed dose response model, for the probability of treatment success at the individual level. For each MCMC sample, the 10th percentile of predicted outcome probability is obtained, i.e. the predicted outcome is better than this value for about 90% of hypothetical future subjects. The resulting posterior distribution demonstrates the uncertainty in the poorest expected outcome, given the MIC and the dose, accounting for the uncertainty contributed by all of the pharmacokinetics, MIC, and the exposure-response model

The use of the PTA as the endpoint for breakpoint determination makes computation relatively simple, but is less efficient than a full dose-response model, due to information loss. Rather than compare each simulated subject’s exposure to a target, with a full dose-response model the posterior predictive or tolerance distributions may be expressed directly in terms of the response variable. From the same simulated exposure-response data, a five-parameter log-logistic model (the correct model) was generated in Stan, using weak prior information, as for the previous example expressed in Fig. 4. The posterior-predicted exposure response function from this model is expressed in Fig. 8. From the final PK model, hypothetical subjects were simulated as above; for each hypothetical subject, the expected response, as the probability of a positive outcome, was obtained by passing the predicted AUC to the exposure-response model. By performing this full operation on each MCMC sample, the resulting posterior predictive and tolerance functions encapsulate the full uncertainty implied by all component models, and are expressed in Fig. 8. The sigmoid form of these functions arises from the forms of both the exposure-response and dose-exposure relationships. While this representation shows the overall patterns, overplotting makes it difficult to draw clear conclusions about any specific features. To highlight the influence of the nominal MIC, the posterior tolerance distribution of the expected outcome, for a series of fixed doses (5, 10 and 25 mg/kg), was generated for each candidate breakpoint. This distribution corresponds to epistemic uncertainty regarding the expected treatment outcome for the poorest 10% of future subjects, which has been abbreviated here as ‘poorest probable outcome’ (PPO), expressed in Fig. 9. Unlike the PTA, which expresses the prediction in terms of a target that has no direct interpretation, this presentation in terms of PPO is directly interpretable in terms of the predicted clinical outcome, given an exposure-response model, and naturally conveys the technical uncertainty of the entire modelling process, including epistemic and aleatoric uncertainties. From Fig. 9, it is clear that while the available information supports the general form of the dose-response relationship, the predictions are clearly meaningfully uncertain, so under these conditions the exact outcome could not be known. The degree of support for any particular candidate breakpoint under these conditions would therefore depend strongly on which uncertainty components were included, and descriptions such as PTA functions derived from point estimates alone may be substantially overconfident.

## Propagation of uncertainty: individual dose optimization

Breakpoint determination involves reasoning about populations. A similar workflow may be applied to evaluate the appropriateness of an antimicrobial dosage regimen for a specific patient or context, given individuating information. Due to the different modelling emphasis, the influence of various uncertainty features can also be reasonably expected to differ.

Suppose that ciprofloxacin is considered for intravenous or oral administration in the clinic, under the previous model, using individual-specific information. From two hypothetical patients, one receiving ciprofloxacin IV and one PO, the plasma concentration of ciprofloxacin was observed at both 30 min and 24 h. These subjects were simulated from the true population distributions of the pharmacokinetic parameters, making them truly exchangeable with the richly-observed subjects. The PK observations were contaminated by the same proportional residual error. These subjects were added to the previous dataset, to simulate the information provided by therapeutic drug monitoring (TDM). This situation emulates a hypothetical case where information is rapidly available to make a single dosing decision via model-informed precision dosing (MIPD).

In this case, these MIPD subjects were simply added to the total dataset, and the parameter estimation conducted again, by the same MCMC procedure. In practice this estimation would probably be conducted sequentially, presuming that the statistical model is generated in advance before the MIPD for the individual is to be done, as in Maier et al. (2020); the single-step process is statistically comparable, in that the MIPD subjects are presumed to be drawn from the same joint population distribution of the PK parameters as the subjects from which the PK model was generated. This exchangeability assumption [74] is practically important but may be problematic due to patient-specific characteristics.

**Fig. 10:**
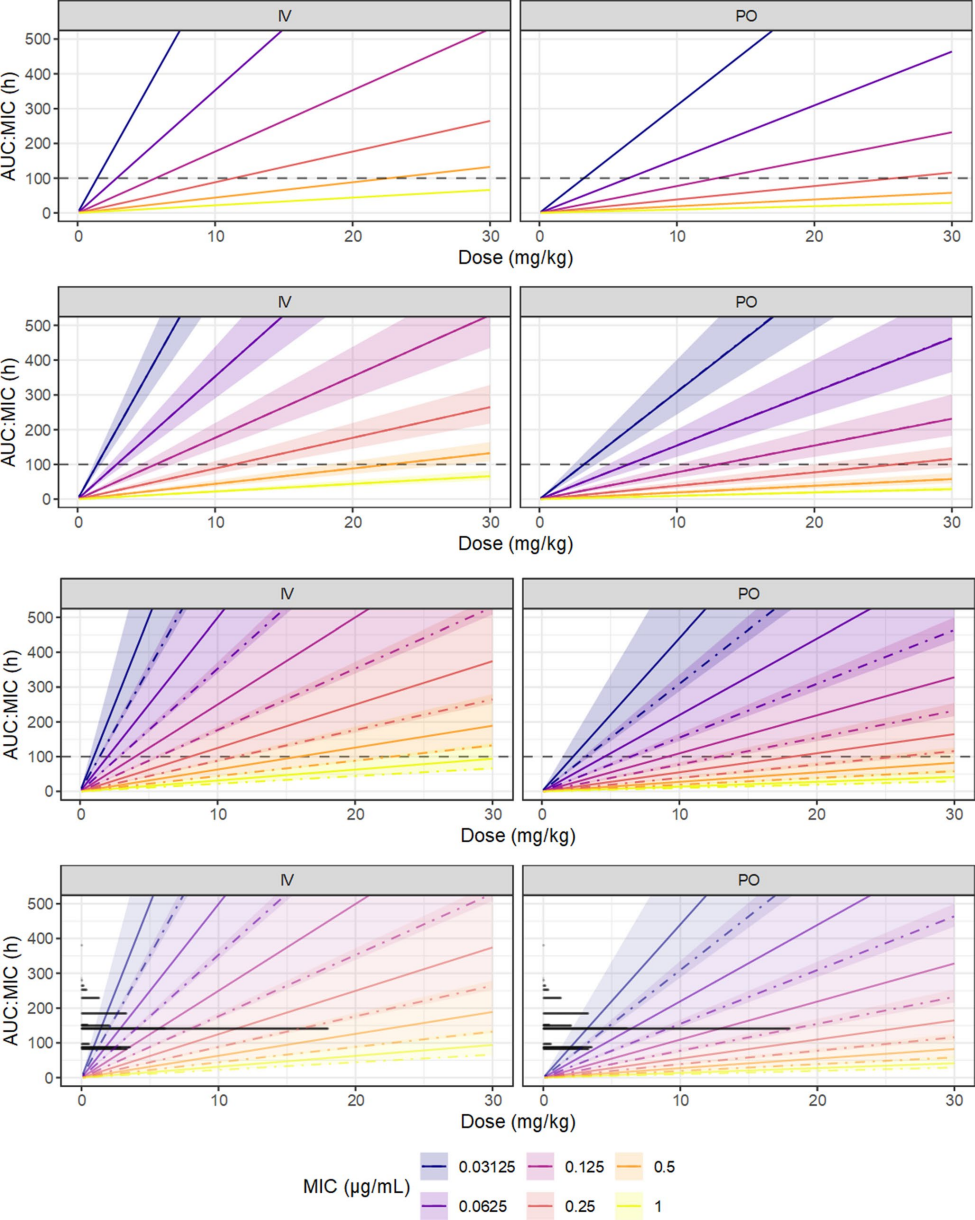
Model-based dose optimization for two hypothetical subjects, one receiving ciprofloxacin by IV bolus and one PO, based on two simulated therapeutic drug monitoring observations for each subject. Posterior-predicted AUC were obtained for individual pharmacokinetic parameters.The solid lines are the posterior median prediction, and the fields the 90% credible regions of the predictions (posterior quantiles). The dashed lines are the corresponding point estimates resulting from ignoring uncertainty (equivalent to the top panel). The horizontal dashed lines indicate the AUC:MIC target. The top panel includes no uncertainty and corresponds to a point estimate. The second panel adds the parameter uncertainty from the PK model. The third panel adds the inherent uncertainty of the MIC. The bottom panel adds the uncertainty in the exposure target, which is represented by its bootstrap distribution as the histogram; note that a handful of bootstrap samples had a value larger than 500h and are not displayed (the full distribution is shown in fig. 7)

**Fig. 11:**
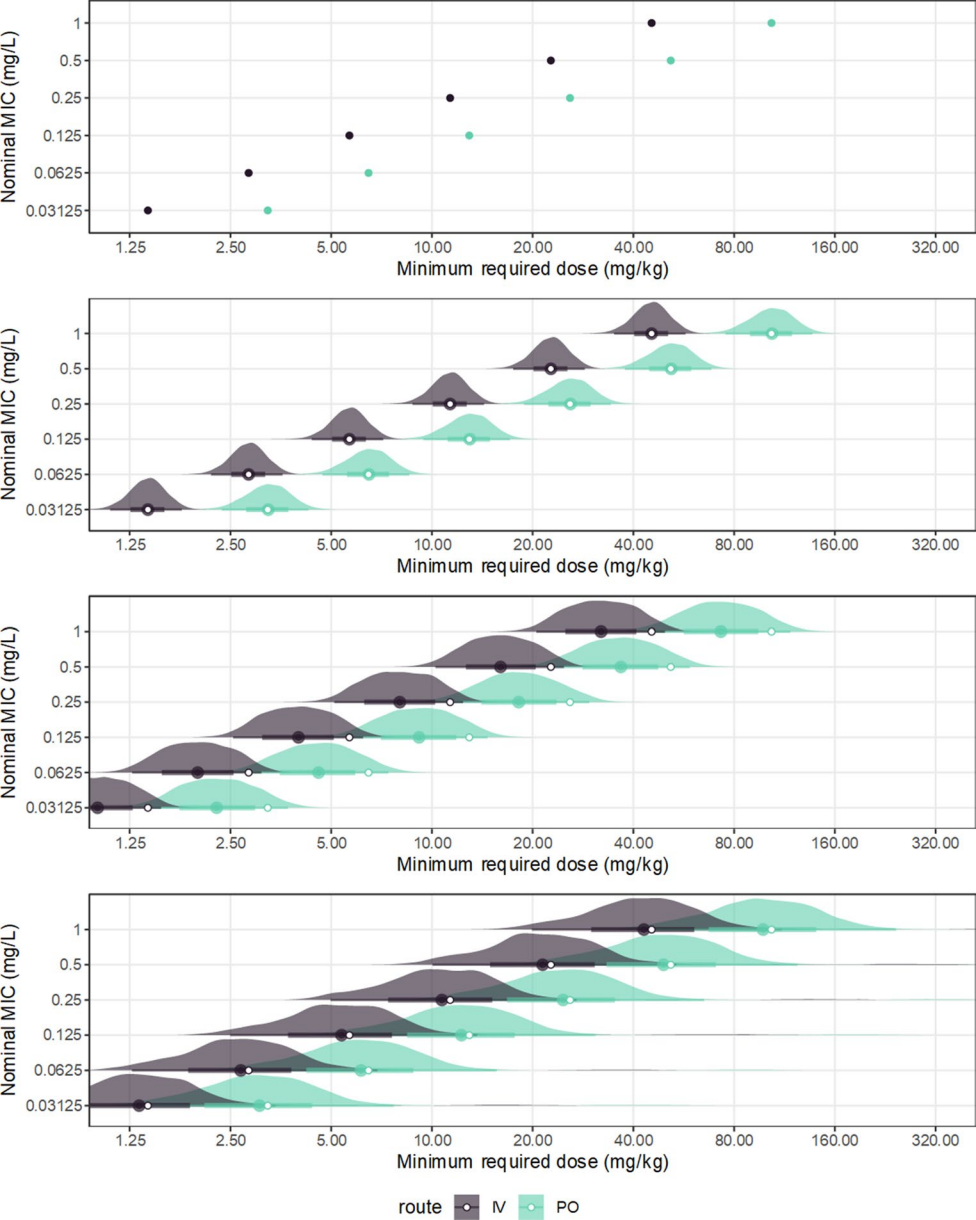
Model-based dose optimization for two hypothetical subjects, one receiving ciprofloxacin by IV bolus and one PO, based on two simulated therapeutic drug monitoring observations for each subject. Posterior predicted AUC were obtained for individual pharmacokinetic parameters. Each panel indicates an estimate of the dose required for that subject to achieve the AUC:MIC target, given the nominal MIC; the AUC:MIC target was set as 100h, except for the bottom panel, which took uncertainty of the target into account. The top panel includes no uncertainty and corresponds to a point estimate. The second panel adds the parameter uncertainty from the PK model. The third panel adds the inherent uncertainty of the MIC. The bottom panel adds the uncertainty in the exposure target. In each case thecentral points are the posterior median. The open points are the corresponding point estimates resulting from ignoring uncertainty (equivalent to the top panel)

For simplicity, the PD target was assumed to be the $\:{AUC}_{\infty\:}:MIC$, which can be easily computed from the clearance and bioavailability. The posterior distributions of the individual PK parameters for the MIPD subjects, emulating what would be learned in a practical case, were extracted from the fitted model; these estimates of the individual pharmacokinetic parameters, for all subjects in the model including these MIPD subjects, are summarized (supplementary Fig. 12). Posterior predictions incorporating various uncertainty sources were generated in sequence. Firstly, only point estimates of the PK parameters (posterior median), the nominal value of the MIC, and a fixed target $\:{AUC}_{\infty\:}:MIC$ (100 h) were used, corresponding to a case where propagation of uncertainty is ignored. Predictions were expressed as the $\:{AUC}_{\infty\:}:MIC$ given the dose and MIC (Fig. 10), and the dose corresponding to $\:{AUC}_{\infty\:}:MIC=100$ for each MIC (Fig. 11). As the inputs are point estimates, the results are also point estimates, so epistemic uncertainty in the results is being ignored, and these predictions overstate the extent of evidence regarding the individuals.

Parameter uncertainty in the PK is included simply using the complete posterior distributions of the individual PK parameters, rather than their posterior medians. This results in full posterior distributions of the predicted $\:{AUC}_{\infty\:}:MIC$ (Fig. 10) and the computed required dose (Fig. 11). This may be extended to incorporate intrinsic uncertainty in the MIC, assuming it was measured on a single twofold dilution series, using the logistic-mollified uniform distribution as earlier. Unlike for the population case, in which the exclusion of the MIC measurement error generated bias but not imprecision in the predictive and quantile functions, here for the individuals the MIC measurement error demonstrates both bias and imprecision, shifting and broadening the posterior distributions relative to the point estimates.

Finally, the parameter uncertainty in the $\:AUC:MIC$ target was incorporated using its bootstrap distribution, as in the population model. A separate bootstrap sample was used as the target $\:AUC:MIC$ at each MCMC sample, resulting in full posterior predictive distributions taking into account the propagation of uncertainty for all inputs simultaneously (Fig. 10). Considering this uncertainty of the target, the approximate posterior distributions of the individual required doses span multiple twofold intervals (Fig. 11). Under these conditions, the capability of the model-based dose optimization to propose the appropriate dose with at least twofold precision was therefore dependent on the value of the PK/PD target being strongly known, likely better than could be provided by a single empirical study. Though conditioning on a single value of the target constrained the span of the posterior distributions to about twofold (Fig. 11), unless this represents actual knowledge rather than a working assumption, this representation is too optimistic. While PK/PD targets may be selected by agreement based on multiple information sources, users of such targets must be aware that individual dosage recommendations are potentially highly sensitive to their uncertainty. The selected target is the sole representation of the relationship between exposure and response, so is doing a lot of heavy lifting; at least, rational decision making must consider a range of plausible values for such a target, especially where the target value is reached by agreement based on subjective consideration of multiple information sources, which are probably noisy.

**Fig. 12:**
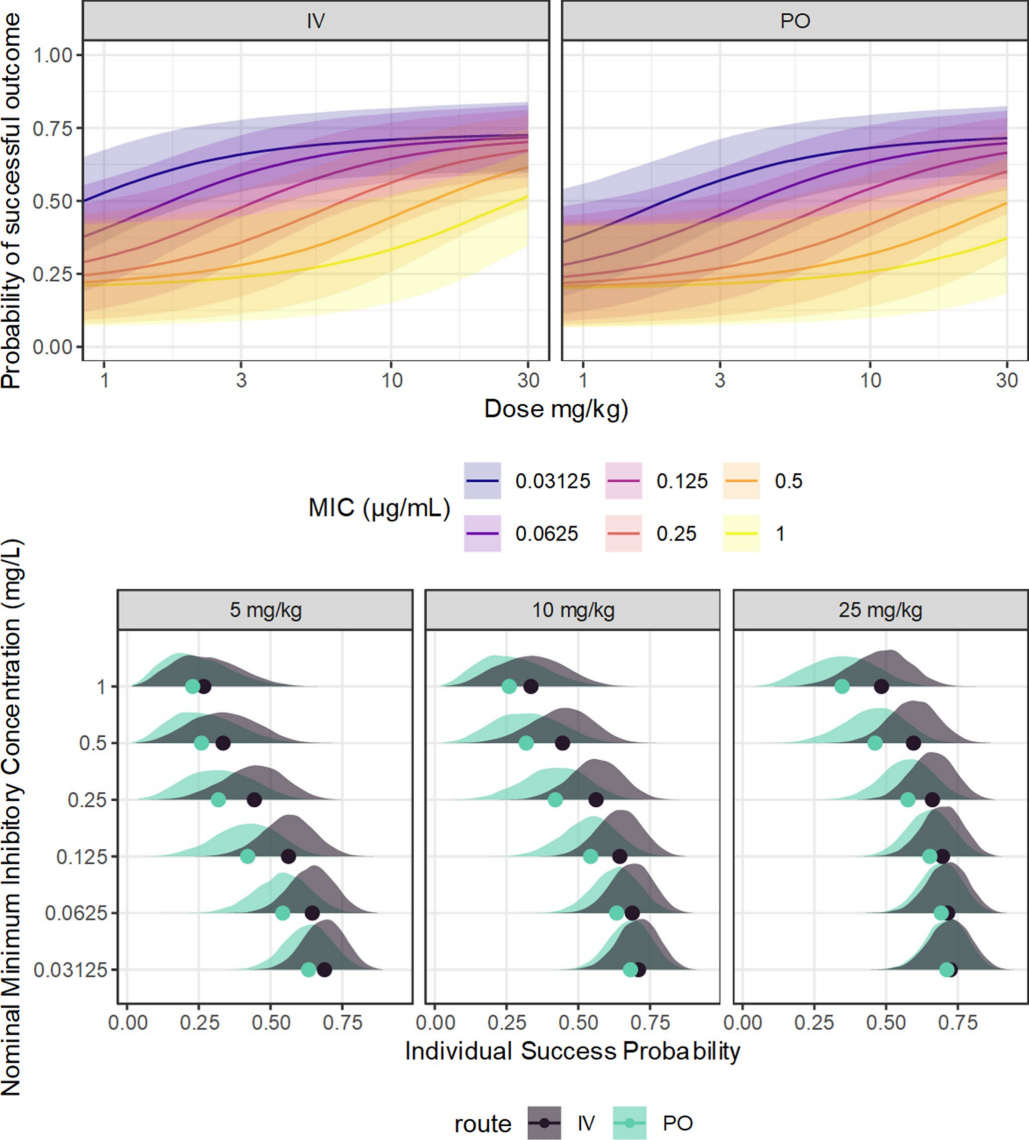
In the upper panel, the approximate posterior distributions of the expected treatment exposure-response relationship, given the dose, for the individuals. The line is the posterior median and the fields the 90% credible regions. Rather than summarize the outcome using an exposure target, the predicted drug exposure for the individual, given their estimated parameters, are passed to the proposed dose-response model, for the probability of treatment success at the individual level. The result is a posterior distribution for the probable outcome given the dose. In the lower panel, the full posterior distributions are expressed conditional on the dose, for a few selected doses. The points are the posterior median

Similarly to the inference regarding breakpoints, a full dose-response model provides more interpretable predictions of the individual outcome. Presuming that the dose-response model is representative for the individual at-hand, the posterior distribution for the expected response for the individual may be obtained by introducing the individual-specific PK predictions to the dose-response model, rather than the population PK predictions as in Fig. 9. The resulting dose-response functions for these individuals, and the full posterior distributions of the expected outcome given the dose for that specific individual, are expressed in Fig. 12. These observations highlight that even with subject-specific PK information, the expected outcome for the individual is meaningfully uncertain, especially for values of the MIC or dose in the middle range.

## Discussion

Quantitative modelling has become a relatively commonplace tool in antimicrobial pharmacology, so any deficiency of the modelling workflow may have broad impact. Though practices in the scientific literature were not surveyed here, in the author’s experience, specific references to technical uncertainty in the antimicrobials literature are rare to non-existent. Though this likely reflects reporting practices throughout the scientific literature, the direct usage of the results for decision-making in antimicrobial pharmacology makes their technical uncertainty particularly important. Quantification of uncertainty in antimicrobial pharmacodynamics workflow would improve the robustness of breakpoints and dosage recommendations, and highlight specific areas where new observations would generate additional knowledge.

A potential objection to this presentation, which was similarly discussed by Colin et al. [11] but is likely even more relevant here, is that practical decision making regarding antimicrobials already presumes uncertainty in these inputs, so uncertainty quantification is unnecessary or uninformative. Regarding breakpoints for example, the decision process is based on an ultimately subjective assessment by a team of reviewers, considering multiple lines of evidence from various sources, rather than a mechanical calculation from the pharmacokinetics and pharmacodynamics [66]. However, without clear quantification of the extent of evidence provided by any specific source, which is an explicitly statistical issue, it may be difficult or impossible for a decision maker to assess accurately, especially when the impact of uncertainties propagates through model features. With careful consideration, direct uncertainty quantification should facilitate, rather than confuse, such decision making.

Predictions expressed solely in terms of point estimates are demonstrably flawed, particularly as they may encourage overconfidence, but the implications of the probability statements illustrated here are important to consider. Statistical uncertainty statements, in general, cannot be taken to represent claims about what is probably true; rather, they express what is implied by the data that has been included in analysis, and the assumptions that have been made. Though estimates and predictions in a Bayesian approach have a convenient interpretation as parameter probabilities, they necessarily reflect biases or misspecification in the underlying design, model and data, and like other inferential statistics should be taken only as local, not global, summaries [75]. While it is helpful to distinguish the posterior probability, as the probable value of a parameter or prediction, from frequentist statements which express data probabilities, such statements are not claims about what is probably true, rather what follows from the data, model, and prior. Conversely, while the generic notion of an error margin seems fairly intuitive, the interpretation of the audience may depend on the exact presentation [76]. Challenges in communication of uncertainty may be exacerbated by common misuse or misinterpretation of hypothesis tests, with the tendency to conflate ‘statistical significance’ with trustworthiness of a point estimate [10].

Replication, and the use of multiple unrelated sources of information to generate inferences or decisions, is clearly an indispensable part of the scientific process, but this essay only considered case studies which considered one data source at a time. Considering measurement errors, for example with the MIC, this is largely unimportant as only the data at hand is relevant, but for processes that rely on external evidence, such as the dose-response relationship, it may be critical. If an exposure target was used in analysis, it would be wise to consider multiple candidate values of that target, but even wiser to aggregate those candidates to come to some general understanding. Unfortunately, exposure targets from decision tree methods or similar techniques that generate a data-based ‘optimal’ threshold, are unsuitable for meta-analysis. Their uncertainty is apparently not widely reported, if at all; without this information, meta-analysis cannot account for the credibility of the individual observations, so comparing their point estimates alone is not meta-analytic and risks vote-counting [77]. As these are functions of the data rather than any proposed data-generating process, some kind of weighted average of thresholds is not even clearly coherent; if they are drawn from differently-shaped exposure-response functions, or from strongly differing distributions of the predictors, their meaning is not very comparable, similarly to the problematic comparison of correlation coefficients [78]. Comprehensive evidence synthesis demands quantification of uncertainty from individual studies.

Uncertainty in terms of the unknown parameters generally receives most attention, and has been the focus here. To generate and evaluate those uncertainties, the form of the model has been held fixed, as if it were perfectly known. In practice, there is always doubt about the correct model, and this lack of knowledge contributes technical uncertainty that should be accounted for [79]. Modelling antimicrobial effects in real-time is difficult, so the normative approach to express drug exposure in antimicrobial pharmacodynamic models has been to collapse a concentration-time function to a summary statistic, removing the time component, and use that as the effect predictor. There are multiple candidates, and it is unclear that any is favourable a priori; the area-under-the-curve, maximum plasma concentration, and time-above-MIC [80] have been popular. Although these may be viewed as special cases of a general underlying PD model [81], determination of the ‘correct’ predictor has generally been a matter of empirical evidence. As the ‘correct’ PD index is not known, the uncertainty associated with this selection should be included in model predictions. In practice, this is likely to be a complicated matter, as information supporting model selection would come from multiple studies.

The contextual nature of many of the important features of these models makes it difficult to draw general conclusions. For example, parameter uncertainties in pharmacokinetic models depend upon the underlying true parameters, the study design including the sample size, the distribution of the doses, routes, times of sampling, and other predictors, and interactions of those factors; it is not clear that any case could be considered representative. What is clear is that in hypothetical cases designed to emulate common presentations in the literature, technical uncertainties were sufficiently large that key decisions would be altered, or not able to be made at all. Similarly, it is not possible to state the extent to which specific findings in the discipline are undermined by unexplored uncertainties, which would require assessment at the study level from original data. It appears reasonable to believe that where specific quantification of uncertainty is not provided, the extent of evidence may be meaningfully overstated, especially where uncertainty propagation is relevant. Without contextual consideration of technical uncertainties, the classical workflow in antimicrobial pharmacology is apparently not a safe basis for decision-making. To ensure that modelling-supported decisions are robust, researchers should ensure that they generate and report uncertainties, while clinicians and other decision-makers should interpret results with reference to their uncertainty. Development of standardizable, practical tools for uncertainty communication in antimicrobial pharmacology is a clear priority.

## Supplementary Information

Below is the link to the electronic supplementary material.

Supplementary Material 1 (PDF 4.10 MB)

## Data Availability

The presentation is based entirely on simulations developed by the author, which emulate the form of published studies where specified. The code supporting the simulations (including the actual data presented in the paper), analyses, and generation of graphics and other outputs is publicly available at https://github.com/APWoodward/antimicrobial_uncertainty.
