# Supplementary material for: Uncertainty undermines the validity of antimicrobial pharmacodynamics
Source: J Pharmacokinet Pharmacodyn. 2026 Mar 2;53(2):13. doi: 10.1007/s10928-026-10023-0 (PMC12953422; doi:10.1007/s10928-026-10023-0)
Supplement: Supplementary file 1 — Supplementary Material 1 (PDF 4.10 MB) [file 10928_2026_10023_MOESM1_ESM.pdf]

Appendix 1

"Uncertainty Undermines the Validity of Antimicrobial Pharmacodynamics"

Journal of Pharmacokinetics and Pharmacodynamics

Andrew P. Woodward[1]

1: Precision One Health Initiative, College of Veterinary Medicine, University of Georgia, Athens, Georgia, USA

andrew.woodward@uga.edu

woodward.andrewp@gmail.com

ORCiD: 0000-0001-5464-9567

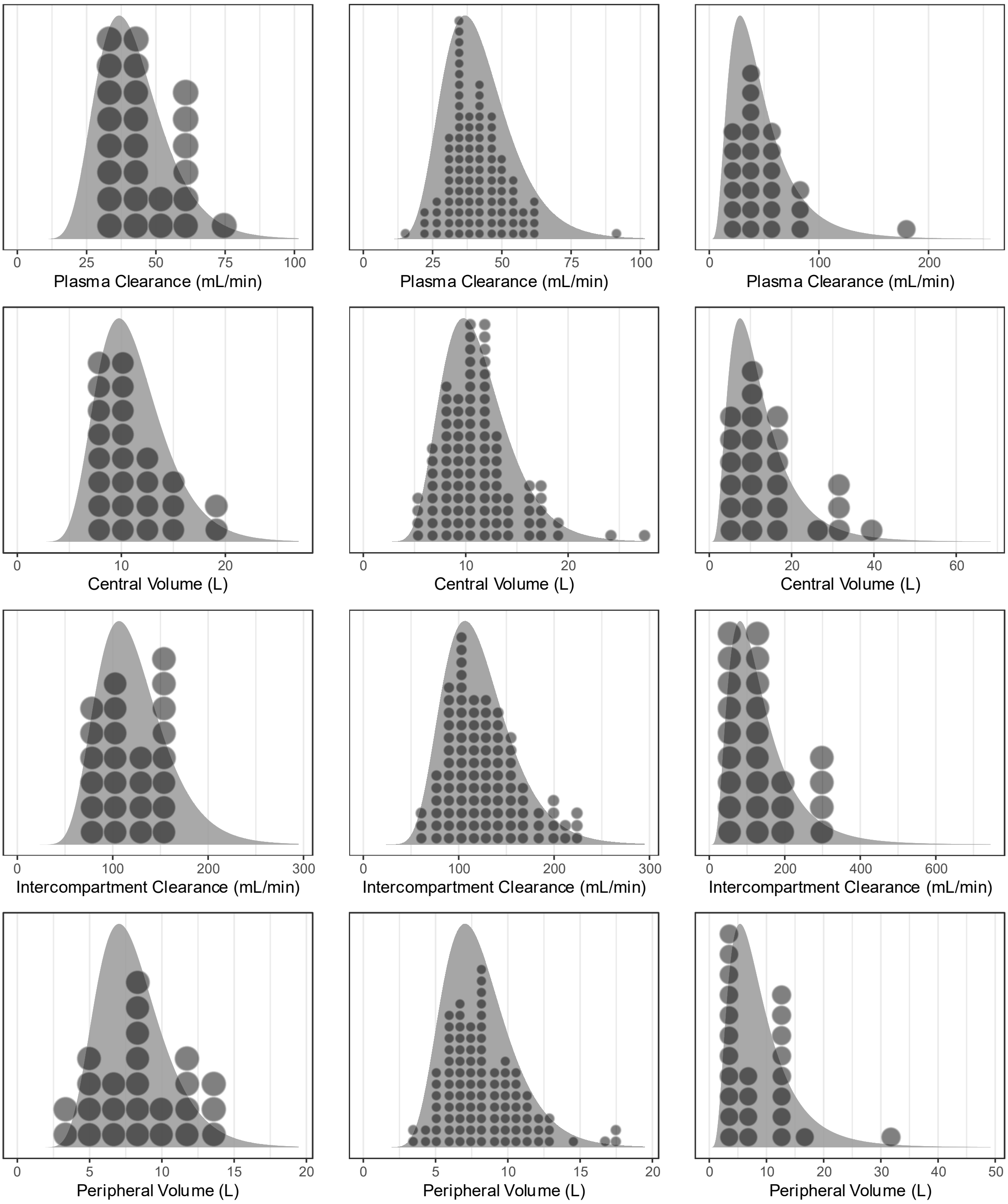

Supplementary figure 1: simulated individual pharmacokinetic parameters (dot histograms) and generating distributions (densities) for amikacin pharmacokinetics, with the true population parameters implemented to emulate those reported by Kato *et. al.* (2017). The first column is for model A (25 subjects, σ: 0.3), the second column is for model B (100 subjects, σ: 0.3), and the third column is for model C (25 subjects, σ: 0.6).

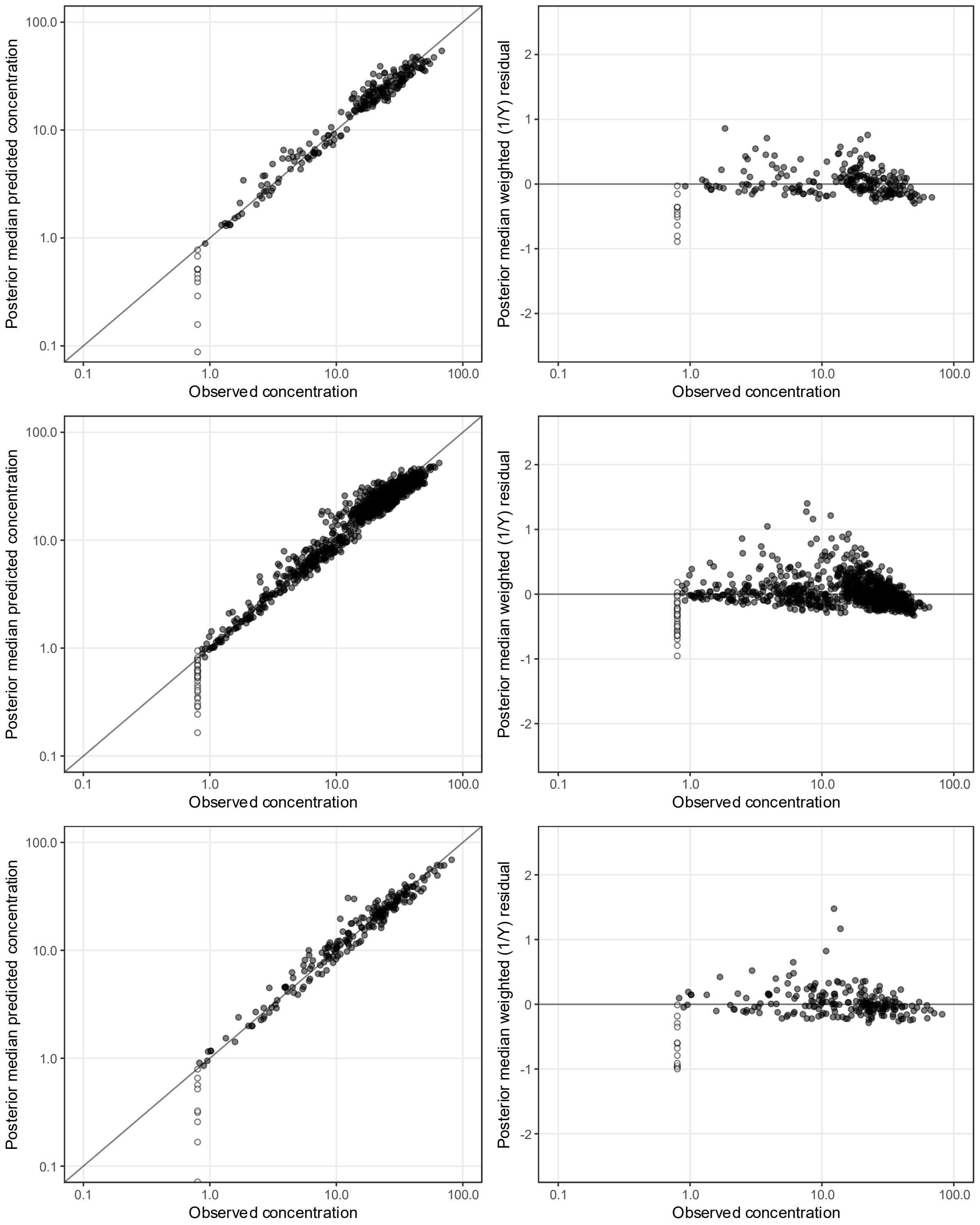

Supplementary figure 2: goodness-of-fit for the models for amikacin pharmacokinetics, based on simulated data. On the left are identity (Y = X) plots and on the right weighted (1/Y) residuals plots, where the predictions are the posterior median. The open points are left-censored observations. The first row for model A had 25 subjects and moderate between-subject variation. The second row for model B had 100 subjects and moderate between-subject variation. The third row for model C had 25 subjects and high between-subject variation.

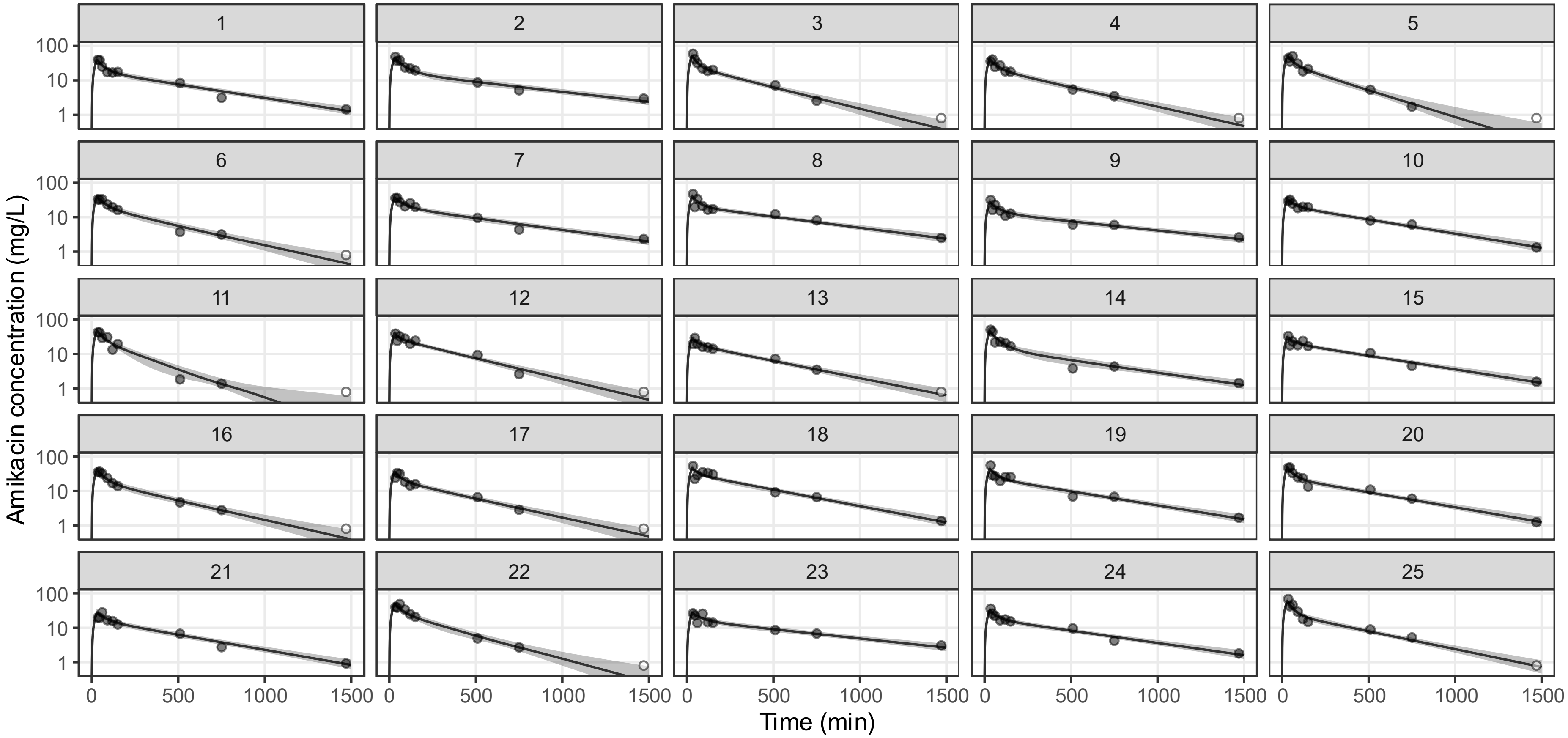

Supplementary figure 3: individual-level predictions (one subject per facet) for amikacin pharmacokinetics, based on simulated data, for model A. The solid line is the posterior median predicted concentration and the grey field its 50% and 90% credible region (the regions are close the posterior median and obscured on this scale). This filled points are the observed concentration and the open points are left-censored observations.

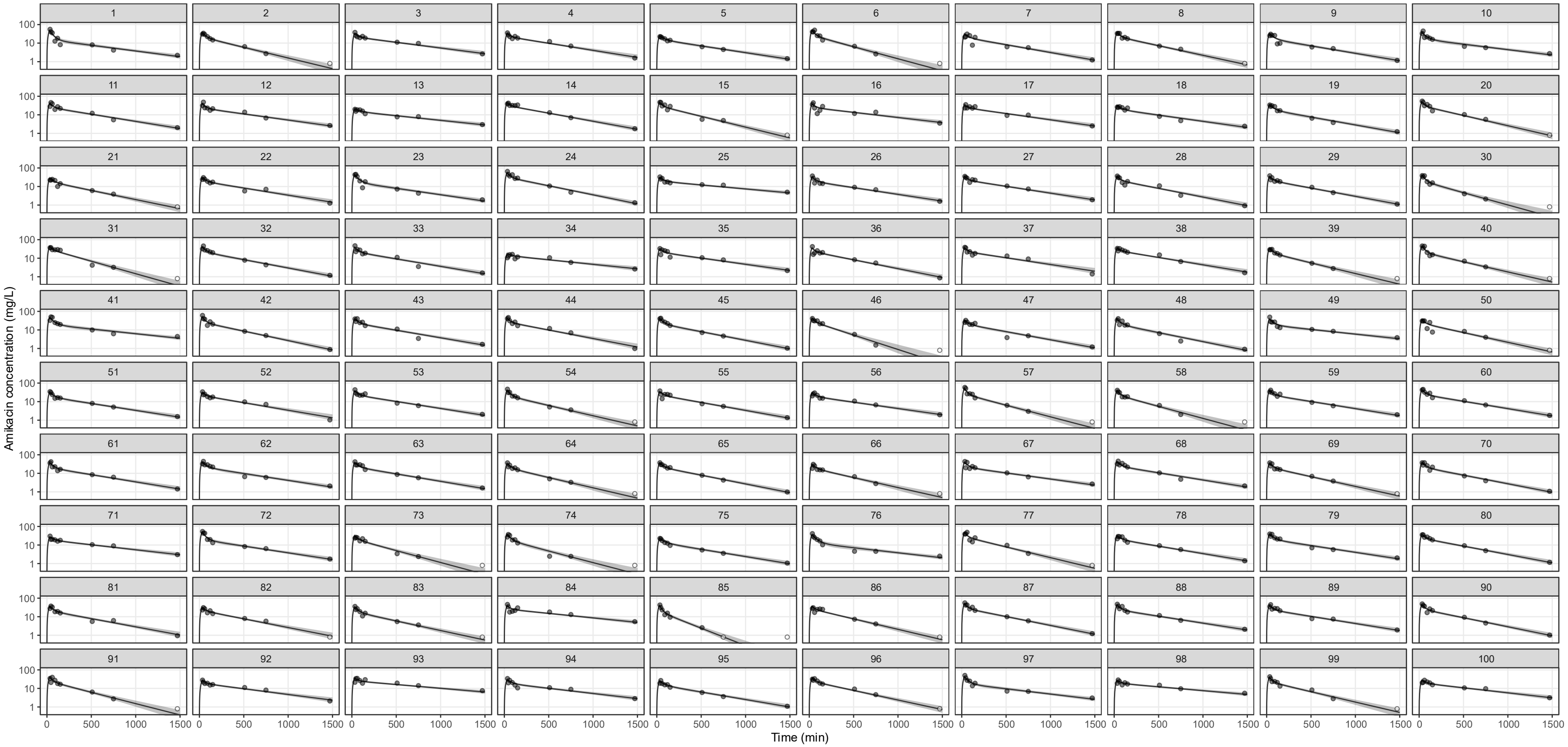

Supplementary figure 4: individual-level predictions (one subject per facet) for amikacin pharmacokinetics, based on simulated data, for model B. The solid line is the posterior median predicted concentration and the grey field its 50% and 90% credible region (the regions are close the posterior median and obscured on this scale). This filled points are the observed concentration and the open points are left-censored observations.

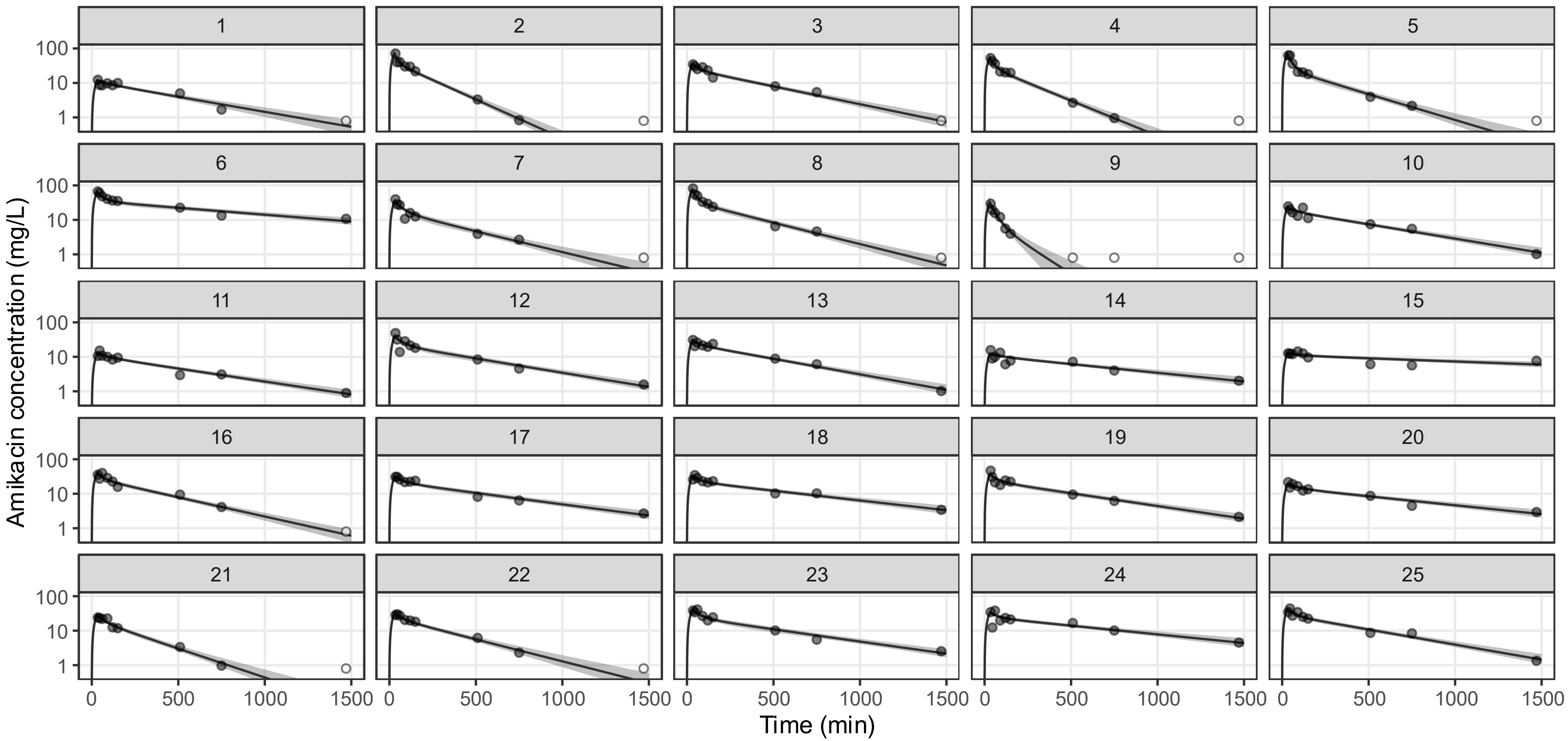

Supplementary figure 5: individual-level predictions (one subject per facet) for amikacin pharmacokinetics, based on simulated data, for model C. The solid line is the posterior median predicted concentration and the grey field its 50% and 90% credible region (the regions are close the posterior median and obscured on this scale). This filled points are the observed concentration and the open points are left-censored observations.

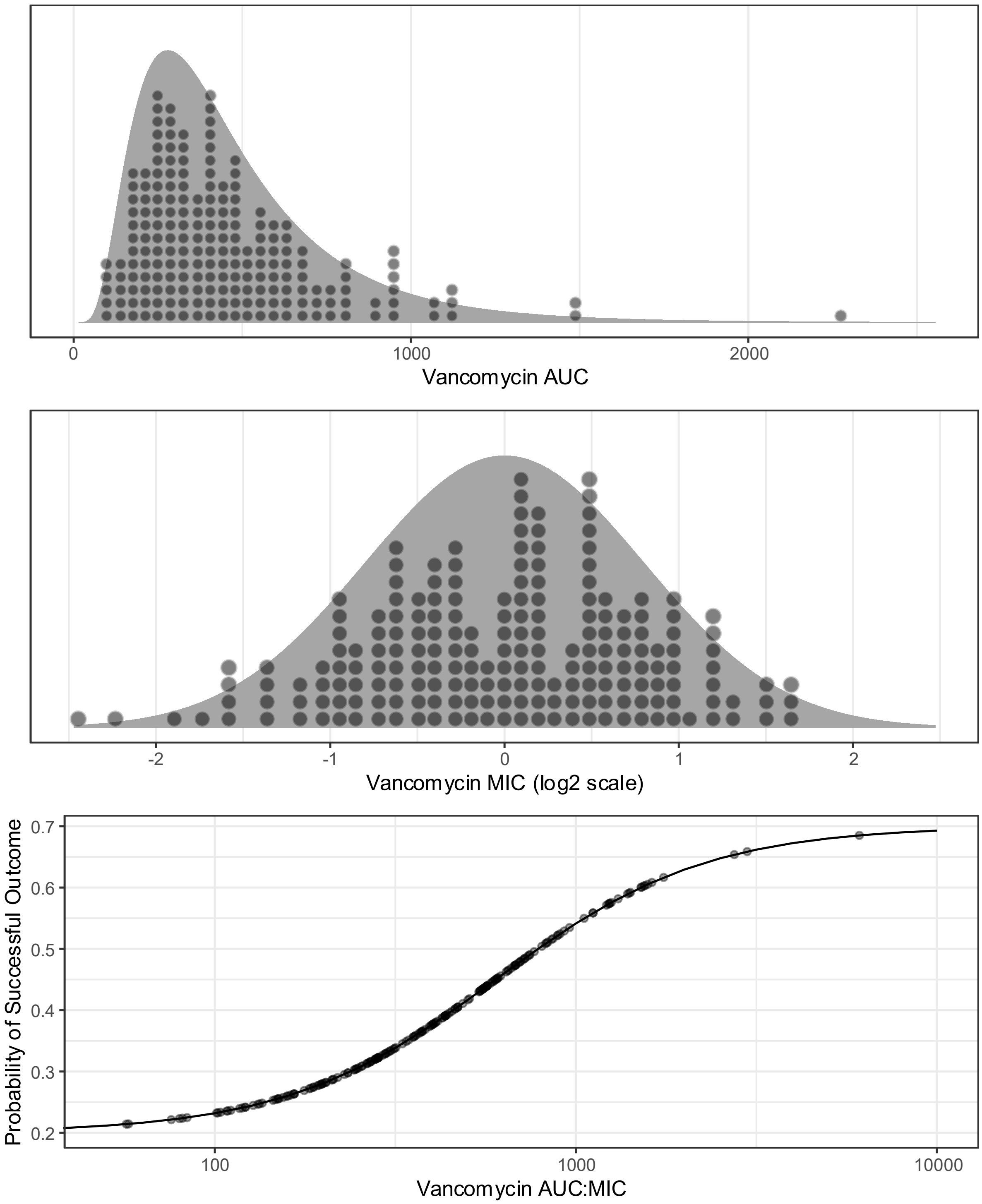

Supplementary figure 6: simulated individual observations of pharmacodynamic variables (dot histograms) and generating distributions (densities) for vancomycin pharmacodynamics. Individual observations are drawn independently for the AUC and MIC, and the individual success probability predicted from the resulting AUC:MIC from a 4-parameter log-logistic model.

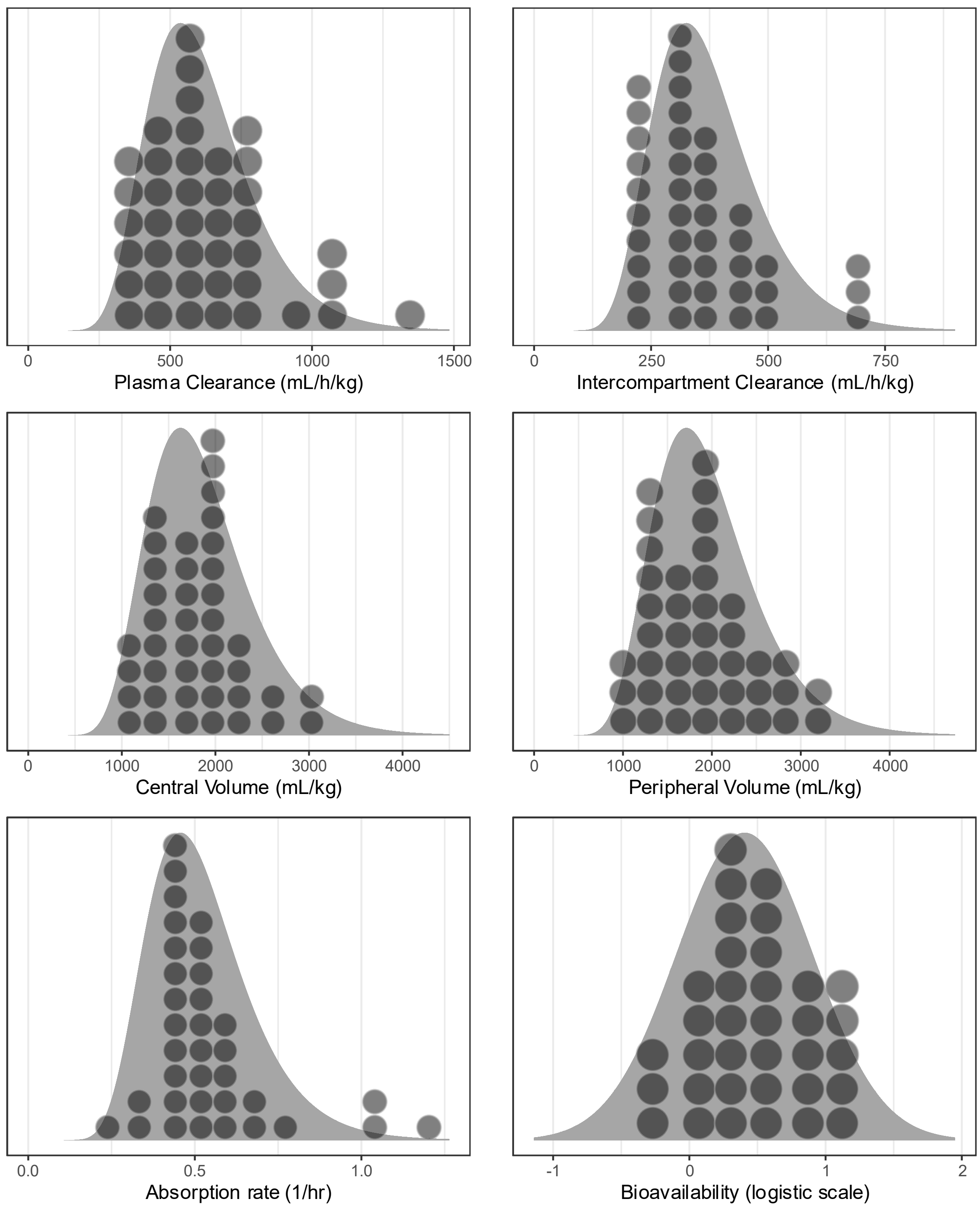

Supplementary figure 7: simulated individual pharmacokinetic parameters (dot histograms) and generating distributions (densities) for ciprofloxacin pharmacokinetics, with the true population parameters implemented to emulate those reported by Papich (2012) and Papich (2017). Hypothetical subjects from both IV and PO phases are included.

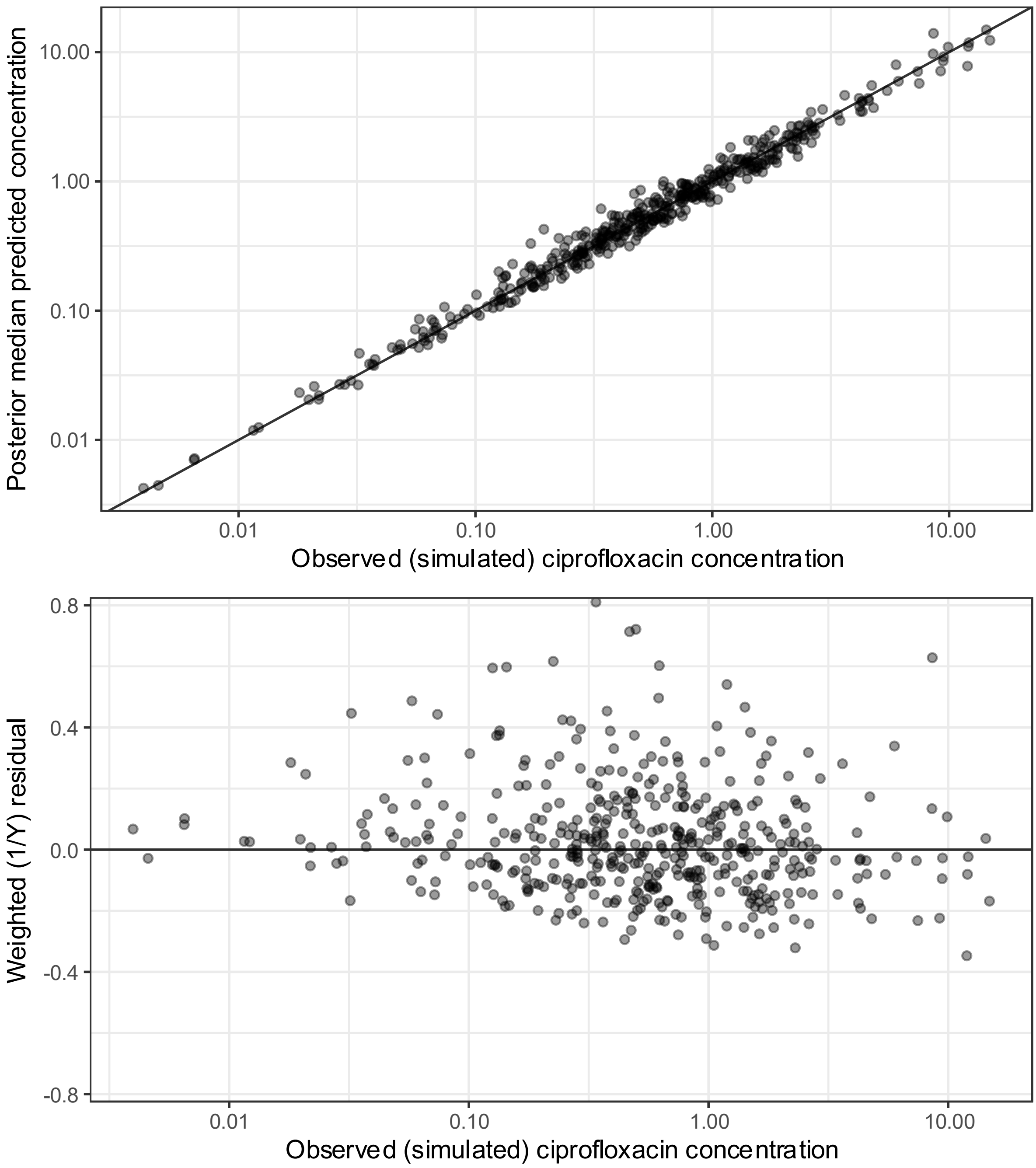

Supplementary figure 8: residual analysis for the ciprofloxacin model. The top is facet is an identity (Y = X) plot and the bottom a weighted residual (1/Y) plot. Predictions and residuals are the posterior median.

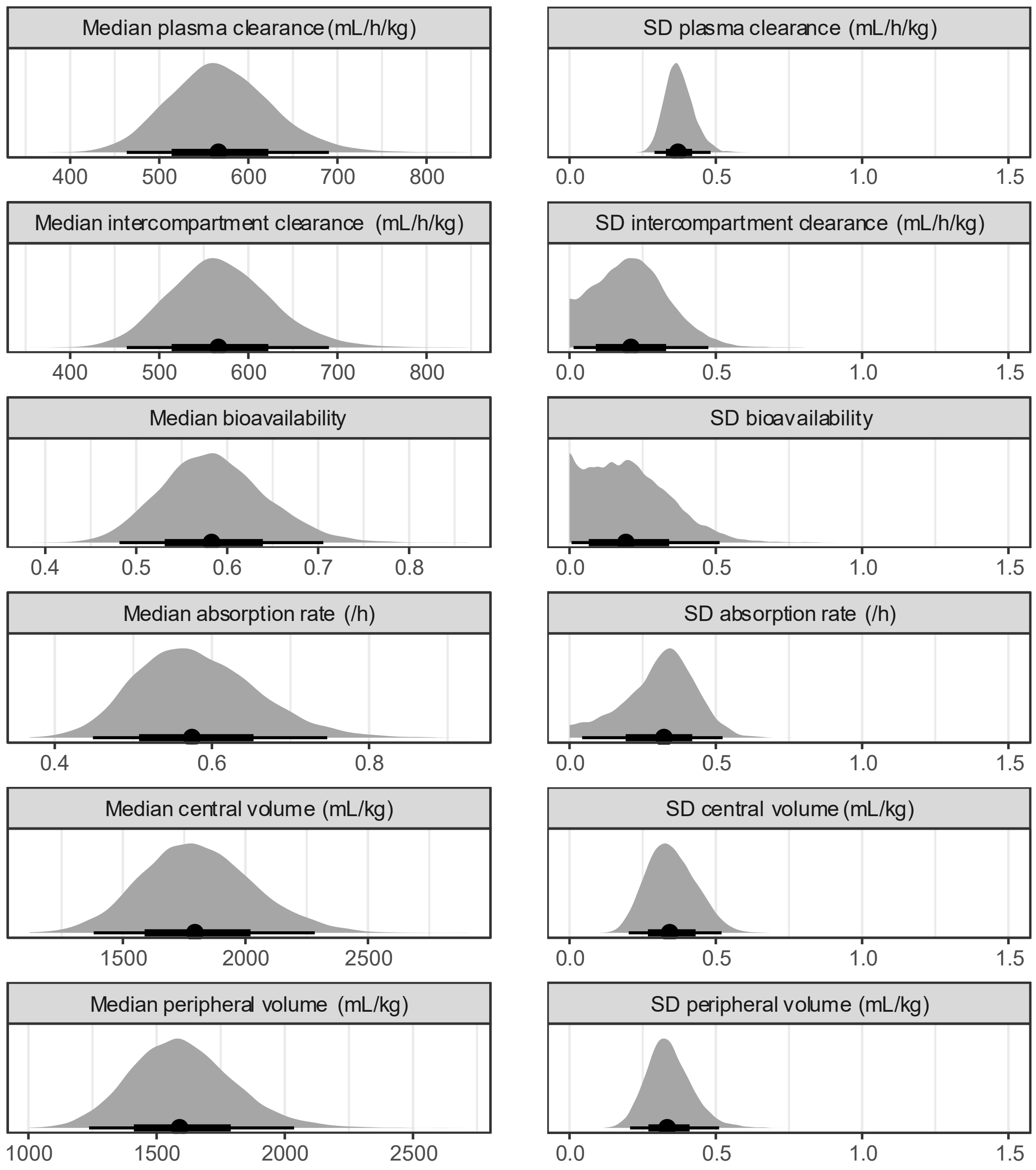

Supplementary figure 9: approximate marginal posterior distributions for the population pharmacokinetic parameters for ciprofloxacin (simulated data), from 6 subjects with IV dosing and 35 subjects with PO dosing. The centre point is the posterior median and the bars the 66% and 95% credible intervals.

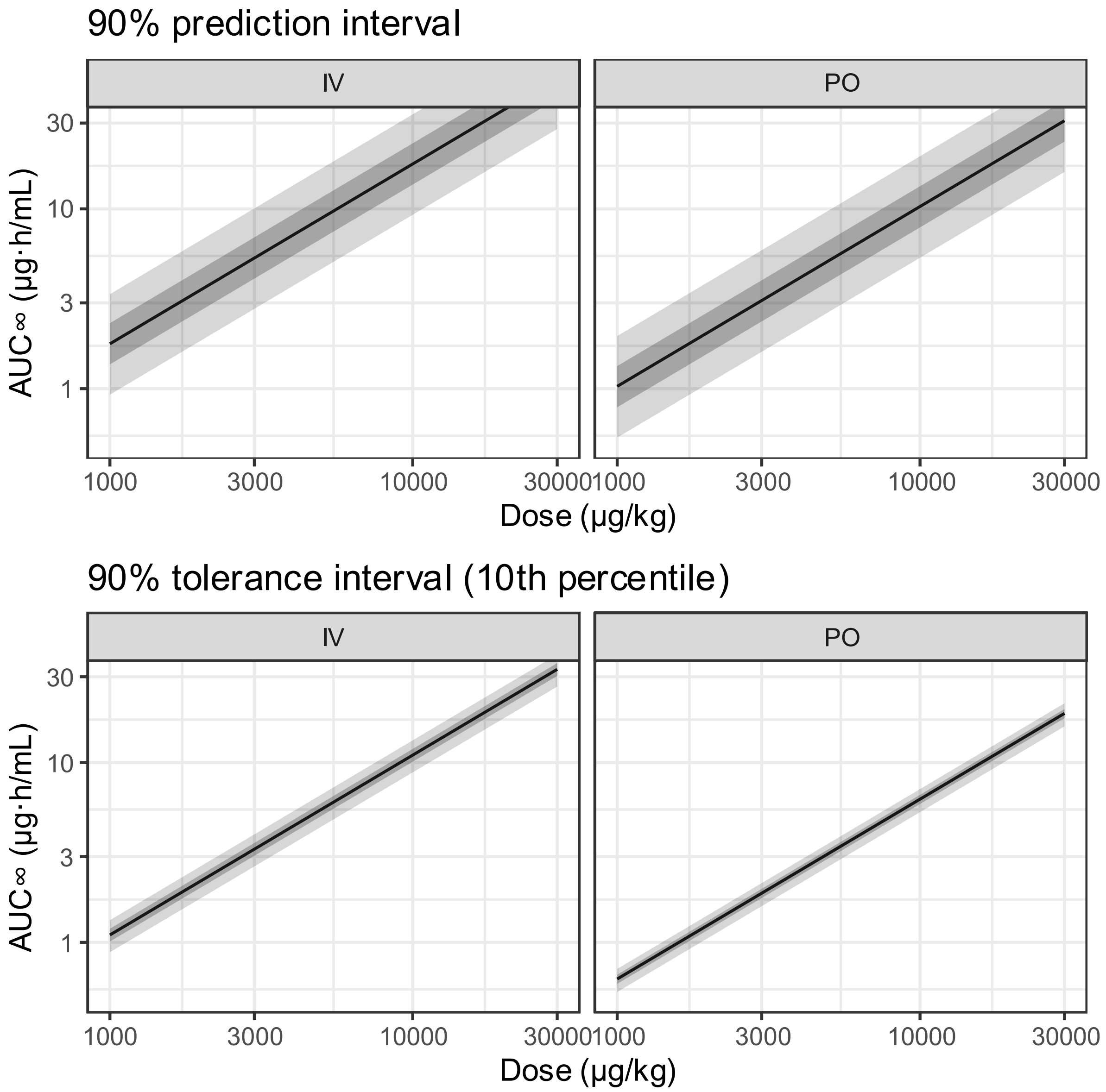

Supplementary figure 10: predictions of ciprofloxacin pharmacokinetics from the population PK model based on simulated data, after IV bolus or oral administration. The top row describes the expected distribution of AUC_∞, given the dose, in hypothetical future subjects. The line is the posterior median, the dark shaded region the 50% predictive interval, and the light region the 90% predictive interval. The predictive distribution takes into account both parameter uncertainty and the between-subject variation; it is composed of one hypothetical subject, simulated from the joint posterior distribution of the PK parameters, for each MCMC sample. The bottom row shows the tolerance distribution, which represents uncertainty in the 10th percentile of hypothetical future subjects, and is generated as the 10th percentile of 1000 simulated subjects at each MCMC sample. The line is the posterior median, and the shaded regions the 50% and 90% credible region for the 10th percentile.

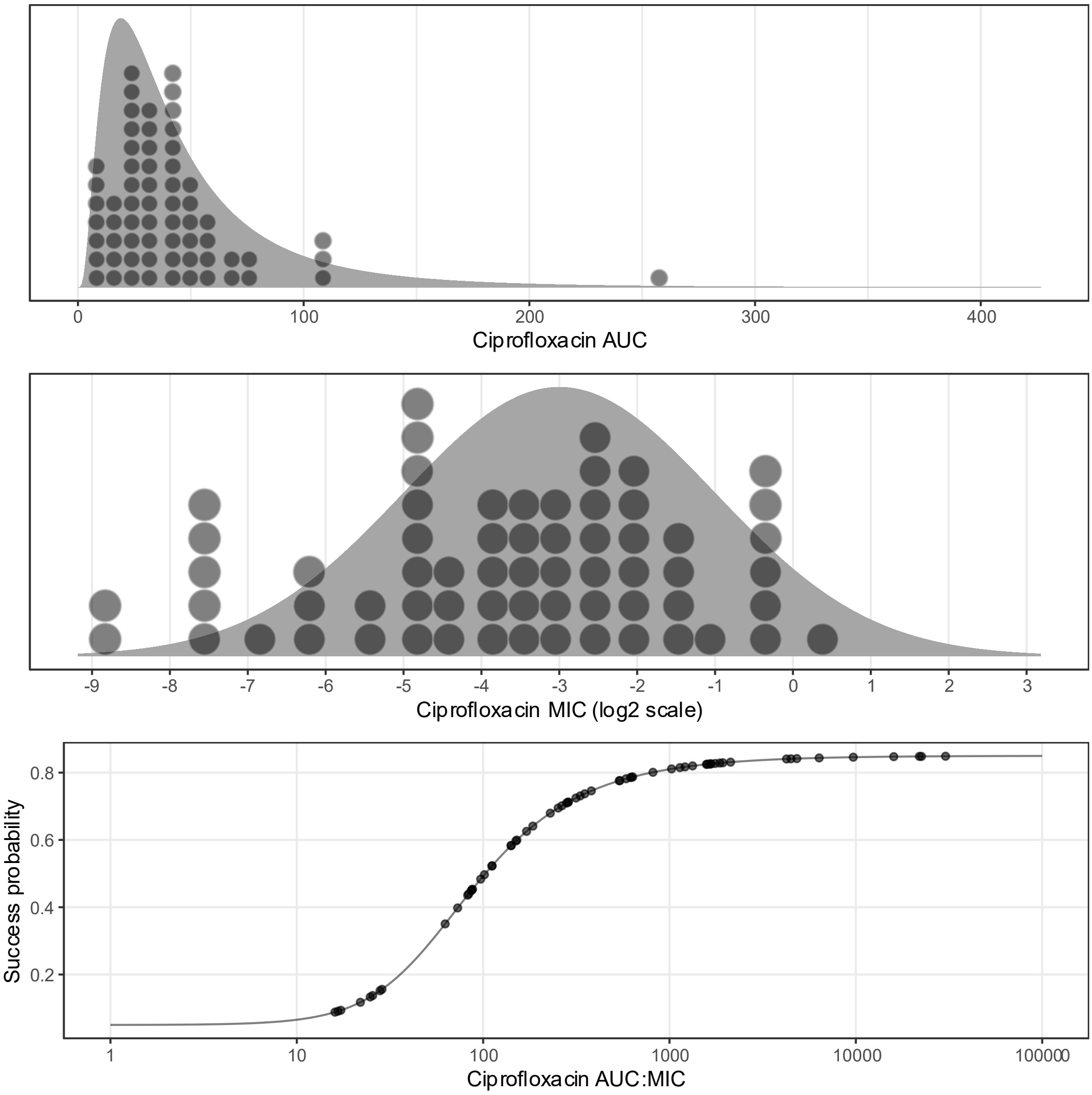

Supplementary figure 11: simulated individual observations of pharmacodynamic variables (dot histograms) and generating distributions (densities) for ciprofloxacin pharmacodynamics, designed to emulate Forrest *et al.* 1993. Individual observations are drawn independently for the AUC and MIC, and the individual success probability predicted from the resulting AUC:MIC from a 5-parameter log-logistic model.

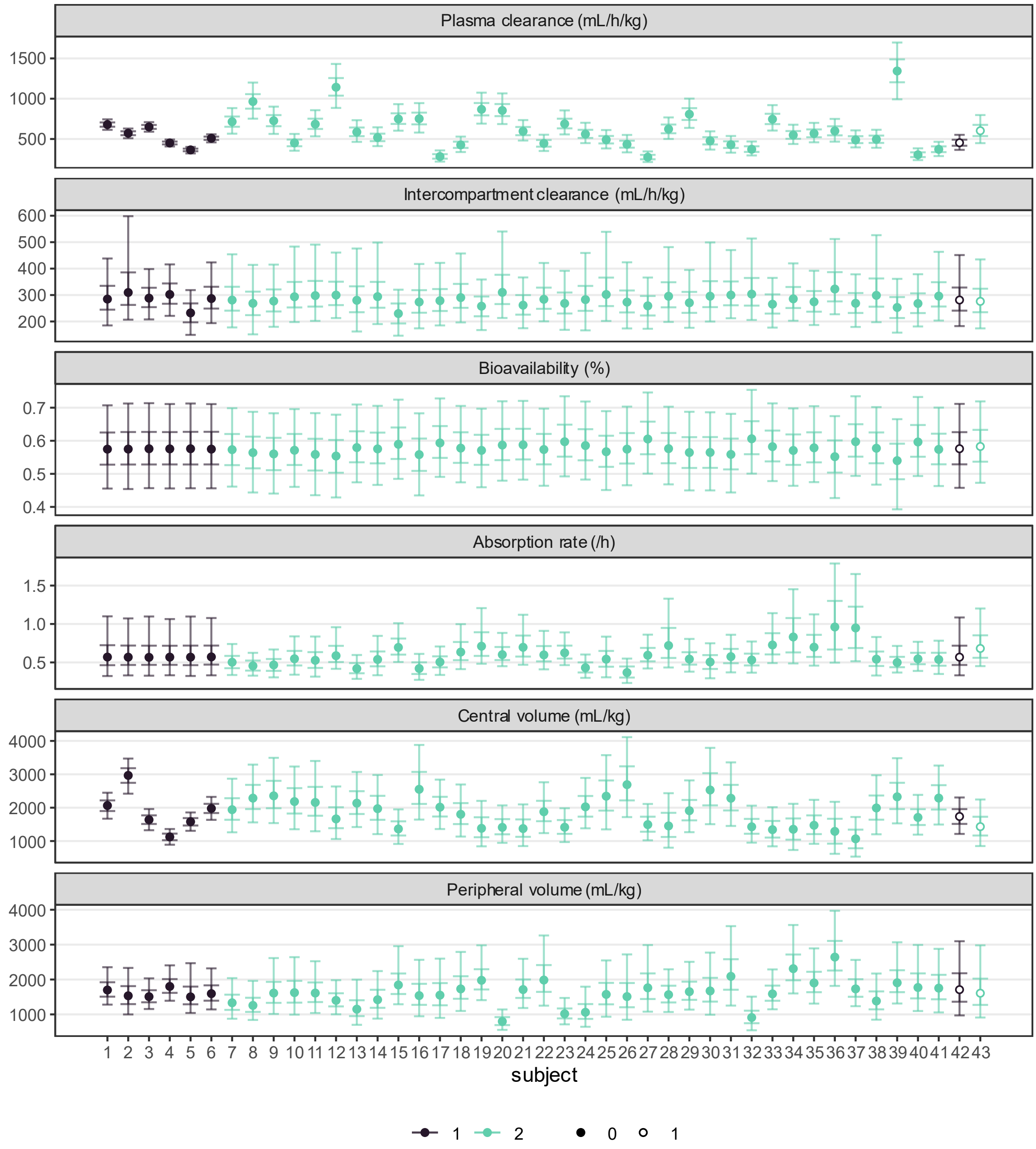

Supplementary figure 12: individual pharmacokinetic parameter estimates from a two-compartment model for ciprofloxacin, using simulated data based on the parameters reported by Papich (2012) and Papich (2017). The closed circles are the marginal posterior median of the individual parameters, and the bars the 50% and 90% credible intervals (posterior quantiles). The open circles indicate the parameter estimates for subjects intended to emulate model-based precision dosing for clinical patients, and are based on only two drug concentration observations for each subject. These are visibly more uncertain than the other subjects due to the relative lack of information. Grey (1) are subjects receiving IV administration, and green (2) PO administration.
